# Indirect comparison of interventions using published randomised trials: systematic review of PDE-5 inhibitors for erectile dysfunction

**DOI:** 10.1186/1471-2490-5-18

**Published:** 2005-12-14

**Authors:** R Andrew Moore, Sheena Derry, Henry J McQuay

**Affiliations:** 1 Pain Research and Nuffield Department of Anaesthetics, University of Oxford, Oxford Radcliffe NHS Trust, The Churchill, Headington, Oxford, OX37LJ, UK

## Abstract

**Background:**

There are no randomised and properly blinded trials directly comparing one PDE-5 inhibitor with another in a normal home setting. Valid indirect comparisons with a common comparator must examine equivalent doses, similar duration, similar populations, with the same outcomes reported in the same way.

**Methods:**

Published randomised, double-blind trials of oral PDE-5 inhibitors for erectile dysfunction were sought from reference lists in previous reviews and electronic searching. Analyses of efficacy and harm were carried out for each treatment, and results compared where there was a common comparator and consistency of outcome reporting, using equivalent doses.

**Results:**

Analysis was limited by differential reporting of outcomes. Sildenafil trials were clinically and geographically more diverse. Tadalafil and vardenafil trials tended to use enriched enrolment. Using all trials, the three interventions were similar for consistently reported efficacy outcomes. Rates of successful intercourse for sildenafil, tadalafil and vardenafil were 65%, 62%, and 59%, with placebo rates of 23–28%. The rates of improved erections were 76%, 75% and 71%, respectively, with placebo rates of 22–24%, and NNTs of 1.9 or 2.0. Reporting of withdrawals was less consistent, but all-cause withdrawals for sildenafil, tadalafil and vardenafil were 8% 13% and 20%. All three drugs were well tolerated, with headache being the most commonly reported event at 13–17%. There were few serious adverse events.

**Conclusion:**

There were differences between trials in outcomes reported, limiting comparisons, and the most useful outcomes were not reported. For common outcomes there was similar efficacy between PDE-5 inhibitors.

## Background

Comparing different interventions for the same condition is often difficult. Large direct comparisons are uncommon, and usually represent only a fraction of the total randomised trial data available. Instead we often have large numbers of randomised trials comparing different interventions with the same or similar comparators, like placebo or an active comparator. These may be direct comparisons, but not the direct comparisons we want.

Indirect comparison of interventions using a common comparator is a valuable alternative because it uses more of the published data [1], and has been done before for analgesics in acute pain [2] and migraine treatments [3]. Even this approach can be devalued because clinical trials in published papers are not consistent in the outcomes they report, or the way they report them [4].

The ideal should be to compare interventions of equivalent intensities or dose, in the same condition, at similar disease severity, using the same outcomes properly reported, over the same period of time. In this study we use the example of published studies of phosphodiesterase-5 (PDE-5) inhibitors for male erectile dysfunction to examine the problems of comparing therapies. PDE-5 inhibitors were chosen because they have been introduced within the past decade, by three different pharmaceutical companies, and in an era of good clinical trial practice. There are no good quality trials directly comparing PDE-5 inhibitors at equivalent doses.

## Methods

We sought randomised trials of three PDE-5 inhibitors (sildenafil, tadalafil, vardenafil), with placebo or active comparator, in men with erectile dysfunction of any causation. Previous systematic reviews [5-9] were used to source trials or trial data, supplemented by electronic searches of PubMed (to June 2005) and the Cochrane Library (issue 1, 2005) using drug names and randomis(z)ed trial.

For inclusion a trial had to be randomised and double blind, use one of the three oral PDE-5 inhibitors in men with erectile dysfunction, be conducted in the home setting, last three weeks or longer, have a minimum of 10 men per group, and report useful information on efficacy or adverse events. Abstracts were read, and potentially useful reports retrieved in full paper copy. Decisions on inclusion or exclusion were made by consensus. No information was taken from posters or abstracts, and studies were read carefully to avoid including duplicate material. Studies were scored for reporting quality using a common method [10] utilising reporting of randomisation, blinding and withdrawals. The maximum score possible was 5 points, and no study could be included with fewer than 2 points.

Information extracted from studies included the number of men studied, the cause of erectile dysfunction, and country where the study was performed. Any type of outcome was initially extracted from the studies, in continuous or dichotomous form, and with any dispersion information available. Outcomes could be reported in tables, in graphs, or in text. The following outcomes were sought particularly:

### Efficacy

• Improved erections ("Has the treatment you have been taking over the past four weeks improved your erections?")

• Erections per week

• Successful attempts at sexual intercourse

• More than 60% or 75% successful

• More than 40% successful

• Final score or change from baseline on question 3 of the International Index of Erectile Function (IIEF) [11] ("Over the past four weeks, when you attempted sexual intercourse, how often were you able to penetrate your partner?")

• Final score or change from baseline on question 4 of the IIEF ("Over the past four weeks, during sexual intercourse, how often were you able to maintain your erection after you had penetrated your partner?")

• Final score or change from baseline on the IIEF erectile function domain score

• Normal erectile function at end of study (IIEF total score of 22 or more out of 30)

### Withdrawal

• All cause

• Lack of efficacy

• Adverse event

### Adverse events

• Patient with at least one adverse event

• Severe (using standard adverse event definitions)

• Serious (using standard adverse event definitions)

• Treatment-related

• Headache

• Dyspepsia

• Flushing

• Nasal congestion or rhinitis

• Visual disturbance

• Myalgia and/or back pain

• Other individual adverse events

Other information sought from trials was the relationship between taking a dose of PDE-5 inhibitor and time of attempted or successful sexual intercourse. The aim was to examine evidence for differences between speed of onset or duration of effect.

Guidelines for quality of reporting of meta-analyses were followed where appropriate [12]. The prior intention was to pool data where there was clinical homogeneity, with similar patients, dose, duration, outcomes, and comparators, but not where numbers of events were small, and random chance could dominate effects of treatment [13].

Clinical trials of PDE-5 inhibitors use both fixed dosing and dose optimisation, in which doses can be increased or decreased within set levels to balance improved efficacy with adverse events. We noted that most dose optimisation schedule studies reported that the majority of patients were on the top dose at the end of the study. We therefore chose to include dose optimisation trials with trials of the top fixed dose. We analysed only licensed doses, as listed in the British National Formulary.

Our intent was to minimise subgroup analysis. There was little prior information that erectile dysfunction from particular causes responds differently to treatment. We used the intention to treat definitions used in the studies (usually number of patients randomised, receiving at least one dose of trial drug, and with at least one post randomisation measurement). When that information was not available we used the numbers of men used as denominators in the trial reports.

Mean results for continuous data were calculated and weighted by the number of men in treatment groups. Homogeneity tests and funnel plots, though commonly used in meta-analysis, were not used here because they have been found to be unreliable [14-16]. Instead clinical homogeneity was examined graphically [17]. Relative benefit (or risk) and number-needed-to-treat (or harm) were calculated with 95% confidence intervals. Relative risk was calculated using a fixed effects model [18], with no statistically significant difference between treatments assumed when the 95% confidence intervals included unity. We added 0.5 to treatment and comparator arms of trials in which at least one arm had no events. Number-needed-to-treat (or harm) was calculated by the method of Cook and Sackett [19] using the pooled number of observations only when there was a statistically significant difference of relative benefit or risk (where the confidence interval did not include 1).

The following terms were used to describe outcomes in terms of benefit, harm or prevention of harm:

• When significantly more beneficial events occurred with PDE-5 inhibitor than with placebo we used the term the number-needed-to-treat to produce one event (NNT).

• When significantly fewer withdrawals or adverse events occurred with PDE-5 inhibitor than with placebo we used the term the number-needed-to-treat to prevent one event (NNTp).

• When significantly more adverse events occurred with PDE-5 inhibitor compared with placebo we used the term the number-needed-to-harm to cause one event (NNH).

The format for presentation of trial details and results was decided prospectively, to show the number of trials and patients on which calculations were based, and either the number of events, or event rates, together with relative risk or benefit and NNT. In this way absolute as well as relative differences would be apparent. Summary data would be shown only where it was from two or more studies, with more than 200 patients, and more than 30 events. No formal testing of difference was planned.

## Results

There were 35 included studies using sildenafil [20-54], treating 7135 men with a mixture of conditions causing erectile dysfunction (Table 1); one study [40] was analysed as two trials. Of the men, 3279 received placebo and 3856 sildenafil, of whom all but 90 took licensed doses of 25 to 100 mg. Optimised dosing with 25–100 mg was most used (2546 men), followed by 100 mg (506) or 50 mg (370). All were placebo-controlled with no active comparator. Quality scores were high, 11 trials scoring 5 of 5 points, 17 scoring 4 points, six 3 points, and two 2 points. One further study [55] used a withdrawal model after successful treatment, and information from the 205 men in this study was not included in this analysis.

**Table 1:** PDE-5 inhibitor trials by condition (percent of total)

**Condition**	**Sildenafil**	**Tadalafil**	**Vardenafil**
Mixed aetiology	67	75	73
Diabetes	14	11	13
Prostatectomy	0.0	15	13
Depression	5.7	0.0	0.0
Spinal cord injury	5.3	0.0	0.0
Multiple sclerosis	3.1	0.0	0.0
Coronary heart disease	2.1	0.0	0.0
Radiotherapy for prostate cancer	1.7	0.0	0.0
Renal failure and haemodialysis	1.0	0.0	0.0
Rectal surgery	0.5	0.0	0.0
Spina bifida	0.5	0.0	0.0

There were eight included studies using tadalafil [56-63], treating 2071 men with a mixture of conditions causing erectile dysfunction (Table 1). Of these, 632 received placebo and 1439 tadalafil, of whom all but 35 took licensed doses of 5 to 20 mg. All were placebo-controlled with no active comparator. Doses of 20 mg (1258) or 10 mg (109) were most frequent, with no dose-optimised studies. Quality scores were high, two trials scoring 5 of 5 points, one scoring 4 points, four 3 points, and one 2 points. One further trial [64] was a population dose-response study and was not included.

There were seven included studies with vardenafil [65-71], treating 3374 men with a mixture of conditions causing erectile dysfunction (Table 1). Of these, 1067 received placebo and 2307 vardenafil, all at licensed doses of 5 to 20 mg. Fixed dosing was most used with 10 mg (809) or 20 mg (698), together with some dose optimised studies of 5–20 mg (382). All were placebo-controlled with no active comparator. Quality scores were high, three trials scoring 5 of 5 points, and four scoring 4 points. One further trial [72] had no placebo group and enriched enrolment and was not included.

Details of the included studies are in additional files 1: conditions, country, treatment, dose, duration and quality score [see Additional file 1]; 2: efficacy outcomes, withdrawals, and adverse events (patients with any adverse event, and severe, serious and treatment-related adverse events) [see Additional file 2]; and 3: details of particular adverse events [see Additional file 3].

## Study reporting

All studies provided background information on participants. Typically men had to have a history of erectile dysfunction of at least three to six months, and the average age of men was generally in the mid 50s or older. Some studies had an enriched enrolment in which previous unsuccessful treatment with a PDE-5 inhibitor was an exclusion criterion. This applied to five of eight tadalafil studies, and six of seven vardenafil studies [see Additional file 1], but none of the sildenafil studies. One vardenafil study [70] included only men previously unresponsive to sildenafil.

Studies almost always documented that PDE-5 inhibitor was to be taken as needed to a maximum of one treatment a day, with the additional instruction for sildenafil and vardenafil that the dose be taken about an hour before intercourse. Some studies gave information on the number of doses actually taken. Many of the dose optimised studies reported the proportion of men on maximum dose at the end of the study, which was always over 50%, and typically 60–80%. Trial duration was typically four to 12 weeks, with 12 weeks the commonest duration.

A number of different conditions causing erectile dysfunction were studied (Table 1). All three PDE-5 inhibitors had a similar proportion of men with mixed aetiology (organic, psychogenic, or mixed) and diabetes. Erectile dysfunction after prostatectomy was studied for tadalafil and vardenafil, but not sildenafil; none of a number of studies of sildenafil after prostatectomy could be included, mainly because they were not randomised. With sildenafil, a variety of additional conditions were studied, including depression, spinal cord injuries, multiple sclerosis, coronary heart disease, radiotherapy for prostate cancer, renal failure and haemodialysis patients, rectal surgery and spina bifida.

Many studies were multicentre, often performed in different countries (Table 2). Europe, North America and Australia accounted for most of the men in the studies for tadalafil and vardenafil. Sildenafil studies in addition were reported from South America, Asia, and Africa.

**Table 2:** Patient numbers in PDE-5 inhibitor trials (by world region)

**Region**	**Sildenafil**	**Tadalafil**	**Vardenafil**
Europe	2040	839	309
North America	2486	546	1658
South America	631	0	0
Asia	1026	0	279
Africa	254	0	0
Australia	63	0	0
World	0	0	448
Europe and North America	217	651	580
Europe and Australia	349	0	0

The percentage of men in trials about whom information was provided for various outcomes is shown in Table 3. Reporting of efficacy was mixed. Almost all trials used the outcome of improved erections, and most used the final score or change from baseline in the erectile function domain of the IIEF. Also commonly reported was the percentage of attempts at sexual intercourse that were successful, usually as an average. Final scores on questions 3 and 4 of the IIEF, or change from baseline, were reported in over 90% of sildenafil trials, but in less than 30% of trials of tadalafil or vardenafil.

**Table 3:** Percentage of men in all trials for whom an outcome is reported

**Outcome**	**Slidenafil n = 7077**	**Tadalafil n = 2036**	**Vardenafil n = 3274**
**Efficacy**			
Improved erections	83	83	100
Mean # erections/week	20	0	0
Successful attempts at SI	47	72	100
More than 60/75% successful	10	0	18
More than 40% successful	0	0	0
Final score IIEF Q3	94	27	26
Mean change IIEF Q3	91	27	26
Final score IIEF Q4	94	27	26
Mean change IIEF Q4	91	27	26
Final score EF Domain	56	72	100
Mean change EF Domain	54	83	77
Normal EF at endpoint	8	56	14
**Withdrawals**			
All-cause	83	65	69
Lack of efficacy	81	72	82
Adverse event	90	83	100
**Adverse events**			
Men with any adverse event	42	40	18
Severe	27	25	35
Serious	38	83	78
Treatment related	55	3	43
Headache	99	90	86
Dyspepsia	79	71	73
Flushing	99	73	73
Nasal congestion/rhinitis	64	35	73
Visual disturbances	89	46	55
Back pain	13	73	9
Myalgia/increased CPK	16	60	18
Flu syndrome	17	30	34
CV events	24	27	33
Limb pain	10	11	0
Fatigue	3	15	0
Priapism	29	0	0
Nausea	14	0	0

Study withdrawal for any cause, or because of lack of efficacy or adverse events was commonly reported. The reporting of men with any adverse event, with severe or serious adverse events according to recognised adverse event reporting criteria, or those adverse events considered treatment related by investigators, was not consistent. Most studies reported specific adverse events only if they occurred in a certain proportion of men, usually between 2% and 5%. As a consequence, some adverse events (headache, dyspepsia, flushing, nasal congestion or rhinitis, or visual disturbance) were consistently reported while others were not.

## Analysis

The first stage was a detailed analysis of outcomes for each intervention, followed by a comparison between interventions where there was sufficient information to make the comparison valid. To make comparisons between different interventions, similar outcomes and similar intensities of intervention have to be compared in similar patients. Only those outcomes with a reasonably high and consistent reporting frequency were available for comparison.

### Individual analyses

#### Sildenafil

Results for continuous efficacy outcomes for sildenafil are shown in Table 4. Average results for percentage successful attempts at intercourse, for the erectile function domain score, and for the change in the erectile function domain score from baseline were consistently available for the optimised dose, and for some trials with fixed dose. Combined data for 50 mg and 100 mg fixed doses and dose optimised regimens are reported (50/100).

**Table 4:** Summary of continuous outcomes for sildenafil, tadalafil and vardenafil

**Sildenafil**				
		**Number of**		
**Outcome**	**Dose**	**Trials**	**Patients**	**Weighted mean**
Percentage successful attempts	placebo	16	1448	23
	50/100	16	1589	65
Erectile function domain score	placebo	23	1870	14.0
	50/100	23	1893	22.0
Erectile function domain change	placebo	23	1870	2.7
	50/100	23	1893	10.1
**Tadalafil**				
		**Number of**		
			
**Outcome**	**Dose**	**Trials**	**Patients**	**Weighted mean**

Percentage successful attempts	placebo	6	388	26
	10/20	6	1047	62
Erectile function domain score	placebo	6	388	14.6
	10/20	6	1047	22.3
Erectile function domain change	placebo	7	459	0.8
	10/20	7	1192	8.4
**Vardenafil**				
		**Number of**		
			
**Outcome**	**Dose**	**Trials**	**Patients**	**Weighted mean**

Percentage successful attempts	placebo	7	996	28
	10/20	7	1789	59
Erectile function domain score	placebo	7	996	14.0
	10/20	7	1789	20.1
Erectile function domain change	placebo	7	996	1.3
	10/20	7	1344	8.3

Results for dichotomous variables of efficacy and adverse events for sildenafil are shown in Table 5. Here data were available for most studies. The analysis combined dose-optimised regimens with 100 mg fixed dose, since the bulk of patients on dose-optimised regimens were on 100 mg.

**Table 5:** Summary of dichotomous outcomes for sildenafil

		**Number of**		**Percent with**			
				
**Outcome**	**Dose (mg)**	**Trials**	**Patients**	**Sildenafil**	**Placebo**	**Relative benefit or risk (95% CI)**	**NNT/NNTp/NNH (95% CI)**
**Efficacy**							
Improved erections	25	5	778	68	27	2.5 (2.1 to 3.0)	2.4 (2.1 to 2.9)
	50	5	781	80	27	3.0 (2.5 to 3.5)	1.9 (1.7 to 2.1)
	100	26	5000	76	23	3.4 (3.1 to 3.6)	1.9 (1.8 to 2.0)
	50/100	29	5467	76	23	3.3 (3.1 to 3.5)	1.9 (1.8 to 2.0)

**Withdrawal**							
All-cause	25	3	522	11	14	0.9 (0.6 to 1.4)	not calculated
	50	4	560	10	14	0.7 (0.4 to 1.2)	not calculated
	100	27	5219	7.8	12	0.6 (0.5 to 0.8)	**22 (16 to 34)**
	50/100	30	5562	8.0	12	0.7 (0.6 to 0.8)	**23 (17 to 37)**
Lack of efficacy	25	3	522	3.0	3.7	0.9 (0.3 to 2.5)	not calculated
	50	3	526	1.8	3.5	0.6 (0.2 to 1.9)	not calculated
	100	27	5119	1.1	4.5	0.3 (0.2 to 0.4)	**24 (20 to 32)**
	50/100	30	5463	1.2	4.4	0.3 (0.2 to 0.4)	**25 (21 to 34)**
Adverse event	25	4	777	1.5	1.3	1.0 (0.4 to 3.0)	not calculated
	50	5	819	1.6	1.2	1.7 (0.7 to 4.4)	not calculated
	100	28	5311	1.4	0.6	1.8 (1.1 to 2.7)	***120 (66 to 520)***
	50/100	31	5787	1.6	0.6	1.7 (1.1 to 2.6)	***120 (67 to 560)***

**Adverse events**							
All cause	50/100	18	2852	50	30	1.6 (1.5 to 1.8)	***4.9 (4.2 to 6.0)***
Serious	50/100	17	2591	2.5	2.4	1.1 (0.6 to 1.7)	not calculated
Headache	50/100	34	6386	17	5.2	3.3 (2.8 to 3.9)	***8.6 (7.6 to 10)***
Dyspepsia	50/100	26	4967	7.8	2.3	3.3 (2.5 to 4.4)	***18 (15 to 23)***
Flushing	50/100	33	6363	13	1.9	6.7 (5.2 to 8.7)	***9.0 (8.1 to 10)***
Rhinitis	50/100	21	4283	5.4	2.1	2.5 (1.8 to 3.5)	***31 (23 to 47)***

NNT is given in standard font, **NNTp in bold**, and ***NNH in bold italic***. No NNT/NNTp/NNH was calculated unless there was a statistically significant difference

**Figure 1:**
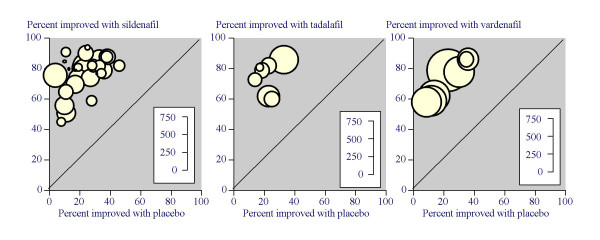
Results of percentage of men with improved erections in individual trials of sildenafil, tadalafil, and vardenafil.

For efficacy, the NNT for improved erections was the same at 1.9 for all doses above 25 mg, demonstrating comparable efficacy and justifying combining those doses. Combining all information on 50 mg and 100 mg with dose optimised regimens (5467 men), improved erections were reported in 76% of men on sildenafil and 23% on placebo (Figure 1). The NNT was 1.9 (95% confidence interval 1.8 to 2.0).

Combining all information on 50 mg and 100 mg with dose optimised regimens, both all cause and lack of efficacy withdrawals reported in about 5600 men, were lower with sildenafil than with placebo, with NNTp values of 23 (17 to 37) and 25 (21 to 34) respectively. Adverse event withdrawals were higher with sildenafil than with placebo, with an NNH of 120 (67 to 560).

Combining all information on 50 mg and 100 mg with dose optimised regimens, particular adverse events were reported in 2600 to 6300 men, and were generally higher with sildenafil than with placebo, except serious adverse events, which were not different. NNH values varied between 4.9 (4.2 to 6.0) for men reporting at least one adverse event (49% of men taking sildenafil), to 18 (15 to 23) for men reporting dyspepsia. Headache was the most commonly reported individual adverse event in 17% of men taking sildenafil, followed by flushing reported in 13% and dyspepsia in 7.8%. The majority of trials reported that adverse events were mostly mild or moderate, and frequently transient.

#### Tadalafil

Results for continuous efficacy outcomes for tadalafil are shown in Table 4. Average results for percentage successful attempts at intercourse, for the erectile function domain score, and for the change in the erectile function domain score from baseline were consistently available for 20 mg fixed dose, but there were 109 men or fewer using 10 mg or less. Combined data for 10 mg and 20 mg fixed doses are reported (10/20).

Results for dichotomous variables of efficacy and adverse events are shown in Table 6. For efficacy, the NNT for improved erections was 1.9 for both the 20 mg fixed dose and combined 10 plus 20 mg analysis. Combining all information on 10 mg and 20 mg, improved erections in 1651 men were reported in 75% of men on tadalafil and 24% on placebo (Figure 1). The NNT was 1.9 (95% confidence interval 1.8 to 2.1).

**Table 6:** Summary of dichotomous outcomes for tadalafil

		**Number of**		**Percent with**			
				
**Outcome**	**Dose (mg)**	**Trials**	**Patients**	**Tadalafil**	**Placebo**	**Relative benefit or risk (95% CI)**	**NNT/NNTp/NNH(95% CI)**
**Efficacy**							
Improved erections	10	2	215	64	23	2.8 (1.9 to 4.2)	2.4 (1.9 to 3.4)
	20	7	1542	76	24	3.1 (2.7 to 3.7)	1.9 (1.7 to 2.1)
	10/20	7	1651	75	24	3.1 (2.6 to 3.7)	1.9 (1.8 to 2.1)

**Withdrawal**							
All-cause	10/20	5	1334	13	19	0.7 (0.5 to 0.9)	**15 (8.8 to 46)**
Lack of efficacy	10/20	6	1435	3.3	7.5	0.5 (0.3 to 0.7)	**24 (14 to 69)**
Adverse event	10/20	7	1657	3.4	1.5	2.3 (1.1 to 5.1)	***52 (29 to 260)***

**Adverse events**							
All cause	10/20	3	590	47	25	1.8 (1.4 to 2.3)	***4.6 (3.4 to 7.2)***
Serious	10/20	7	1655	1.2	1.1	1.0 (0.4 to 2.8)	not calculated
Headache	10/20	7	1810	13	3.4	3.5 (2.2 to 5.4)	***11 (8.5 to 14)***
Dyspepsia	10/20	6	1401	10	0.2	12 (4.3 to 35)	***11 (8.8 to 14)***
Flushing	10/20	6	1530	4.8	0.2	7.2 (2.5 to 20)	***24 (18 to 38)***
Rhinitis	10/20	2	712	3.1	0.5	4.5 (0.8 to 24)	not calculated

NNT is given in standard font, **NNTp in bold**, and ***NNH in bold italic***. No NNT/NNTp/NNH was calculated unless there was a statistically significant difference

Combining all information on 10 mg and 20 mg, both all cause and lack of efficacy withdrawals reported in about 1400 men, were lower with tadalafil than with placebo, with NNTp values of 15 (8.8 to 46) and 24 (14 to 69) respectively. Adverse event withdrawals were higher with tadalafil than with placebo, with an NNH of 52 (29 to 260).

Combining all information on 10 mg and 20 mg, particular adverse events were reported in 600 to 1800 men, and were generally higher with tadalafil than with placebo, except serious adverse events, which were not different, and rhinitis, where there were few events. NNH values varied between 4.6 (3.4 to 7.2) for men reporting at least one adverse event (47% of men taking tadalafil), to 24 (18 to 38) for men reporting dyspepsia. Headache was the most commonly reported individual adverse event in 13% of men taking tadalafil, followed by dyspepsia reported in 10% and flushing in 4.8%. The majority of trials reported that adverse events were mostly mild or moderate, and frequently transient.

#### Vardenafil

Results for continuous efficacy outcomes for vardenafil are shown in Table 4. Average results for percentage successful attempts at intercourse, for the erectile function domain score, and for the change in the erectile function domain score from baseline were consistently available for the optimised dose, and for some trials with fixed dose. Combined data for 10 mg and 20 mg fixed doses and dose optimised regimens are reported (10/20).

Results for dichotomous variables of efficacy and adverse events are shown in Table 7. The analysis combined dose optimised regimens with the 20 mg fixed dose, since the bulk of patients on dose optimised regimens were taking 20 mg.

**Table 7:** Summary of dichotomous outcomes for vardenafil

		**Number of**		**Percent with**			
				
**Outcome**	**Dose (mg)**	**Trials**	**Patients**	**Vardenafil**	**Placebo**	**Relative benefit or risk (95% CI)**	**NNT/NNTp/NNH(95%CI)**
**Efficacy**							
Improved erections	5	3	833	62	27	2.3 (1.9 to 2.7)	2.9 (2.4 to 3.5)
	10	5	1401	68	21	3.3 (2.9 to 3.9)	2.1 (1.9 to 2.3)
	20	7	2147	73	22	3.4 (3.0 to 3.8)	2.0 (1.8 to 2.1)
	10/20	7	2856	71	22	3.3 (3.0 to 3.8)	2.0 (1.9 to 2.2)

**Withdrawal**							
All-cause	10	3	812	4.2	2.5	1.7 (0.8 to 3.7)	not calculated
	20	5	1623	18	32	0.6 (0.5 to 0.7)	**6.9 (5.4 to 9.7)**
	10/20	5	2061	20	32	0.6 (0.5 to 0.6)	**7.7 (6.0 to 11)**
Lack of efficacy	5	2	505	11	18	0.6 (0.4 to 0.9)	**14 (7.5 to 85)**
	10	4	1084	4	15	0.3 (0.2 to 0.4)	**9.3 (7.1 to 14)**
	20	6	1831	4	13	0.3 (0.2 to 0.4)	**11 (8.7 to 15)**
	10/20	6	2320	4	12	0.3 (0.2 to 0.4)	**11 (9.0 to 16)**
Adverse event	5	3	812	4.2	2.5	1.7 (0.8 to 3.7)	not calculated
	10	5	1395	2.8	1.9	1.5 (0.7 to 3.0)	not calculated
	20	7	2161	3.6	1.8	2.1 (1.2 to 5.3)	***54 (31 to 210)***
	10/20	7	2868	3.3	1.8	1.8 (1.1 to 3.0)	***65 (37 to 250)***

**Adverse events**							
All cause	10/20				insufficient data		
Severe	10/20	3	1096	2.7	2.2	1.2 (0.5 to 2.8)	not calculated
Serious	10/20	5	1984	2.2	3.2	0.7 (0.4 to 1.2)	not calculated
Headache	10/20	6	2411	15	4.1	3.4 (2.4 to 4.8)	***9.6 (7.9 to 12)***
Dyspepsia	10/20	5	1972	3.8	0.3	7.3 (2.4 to 22)	***31 (22 to 48)***
Flushing	10/20	5	1984	13	0.8	13 (6.3 to 27)	***8.0 (6.9 to 9.6)***
Rhinitis	10/20	5	2212	7.9	3.6	2.2 (1.5 to 3.4)	***23 (16 to 42)***

NNT is given in standard font, **NNTp in bold**, and ***NNH in bold italic***. No NNT/NNTp/NNH was calculated unless there was a statistically significant difference

For efficacy, the NNT for improved erections was the same at 2.0 for all doses above 5 mg, demonstrating comparable efficacy and justifying combining those doses. Combining all information on 10 mg and 20 mg with dose optimised regimens, improved erections in 2856 men were reported in 71% of men on vardenafil and 22% on placebo (Figure 1). The NNT was 2.0 (95% confidence interval 1.9 to 2.2).

Combining all information on 10 mg and 20 mg with dose optimised regimens, both all cause and lack of efficacy withdrawals reported in about 500 to 2300 men, were lower with vardenafil than with placebo, with NNTp values of 7.7 (6.0 to 11) and 11 (9.0 to 16) respectively. Adverse event withdrawals, reported in about 2800 men, were higher with vardenafil than with placebo, with an NNH of 65 (37 to 250).

Combining all information on 10 mg and 20 mg with dose optimised regimens, adverse event outcomes were reported in 1000 to 2400 men, and were generally higher with vardenafil than with placebo, except serious adverse events, which were not different, and men with at least one adverse event, which was not commonly reported in these trials. The lowest (worst) NNH was 8.0 (6.9 to 9.6) for men reporting flushing. Headache was the most commonly reported individual adverse event in 15% of men taking vardenafil, followed by flushing reported in 13% and rhinitis in 7.9%. The majority of trials reported that adverse events were mostly mild or moderate, and frequently transient.

### Comparing different treatments

**Figure 2:**
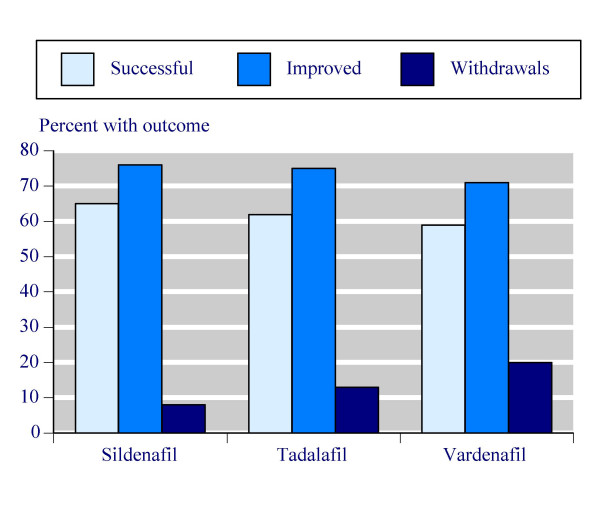
Summary of percentage of successful attempts at intercourse (Successful), percentage of men with improved erections (Improved), and of all-cause withdrawals (Withdrawals) for top doses of sildenafil, tadalafil and vardenafil.

Continuous outcomes are compared in Table 8, which documents the number of trials and patients for which the consistently reported outcomes were available, together with the weighted mean result for both placebo and the top doses plus dose optimised regimens of individual PDE-5 inhibitors. There was remarkable consistency. For instance, the percentage of successful attempts at intercourse with placebo varied narrowly between 23% and 28%. The three PDE-5 inhibitors had success rates of 65% for sildenafil, 62% for tadalafil and 59% for vardenafil (Figure 2). The final erectile function domain score, and change from baseline were highly consistent for both placebo and PDE-5 inhibitors.

**Table 8:** Comparison of continuous efficacy results for sildenafil, tadalafil, and vardenafil

	**Sildenafil (50/100 mg)**				**Tadalafil (10/20 mg)**				**Vardenafil (10/20 mg)**			
		**Number of**				**Number of**				**Number of**		
									
**Outcome**	**Dose**	**Trials**	**Patients**	**Weighted mean**	**Dose**	**Trials**	**Patients**	**Weighted mean**	**Dose**	**Trials**	**Patients**	**Weighted mean**
Percentage successful attempts	placebo	16	1448	23	placebo	6	388	26	placebo	7	996	28
	50/100	16	1589	65	10/20	6	1047	62	10/20	7	1789	59

Erectile function domain score	placebo	23	1870	14.0	placebo	6	388	14.6	placebo	7	996	14.0
	50/100	23	1893	22.0	10/20	6	1047	22.3	10/20	7	1789	20.1

EF domain change	placebo	23	1870	2.7	placebo	7	459	0.8	placebo	7	996	1.3
	50/100	23	1893	10.1	10/20	7	1192	8.4	10/20	7	1344	8.3

Dichotomous outcomes are compared in Table 9, documenting the number of trials and patients for which the consistently reported outcomes were available, together with event rates for PDE-5 inhibitor and placebo, and the NNT/NNTp/NNH values obtained. For efficacy, using improved erections as the outcome, there was a high degree of consistency for placebo with rates between 22% and 24%. The three PDE-5 inhibitors had event rates of 76% for sildenafil, 75% for tadalafil and 71% for vardenafil (Figure 2). NNTs for all three PDE-5 inhibitors were 1.9 or 2.0.

**Table 9:** Comparison of dichotomous efficacy results for sildenafil, tadalafil, and vardenafil

	**Sildenafil (50/100 mg)**					**Tadalafil (10/20 mg)**					**Vardenafil (10/20 mg)**				
	**Number of**		**Percent with**			**Number of**		**Percent with**			**Number of**		**Percent with**		
						
**Outcome**	**Trials**	**Patients**	**Sildenafil**	**Placebo**	**NNT/NNTp/NNH (95% CI)**	**Trials**	**Patients**	**Tadalafil**	**Placebo**	**NNT/NNTp/NNH (95% CI)**	**Trials**	**Patients**	**Vardenafil**	**Placebo**	**NNT/NNTp/NNH (95% CI)**
**Efficacy**															
Improved erections	29	5467	76	23	1.9 (1.8 to 2.0)	7	1651	75	24	1.9 (1.8 to 2.1)	7	2856	71	22	2.0 (1.9 to 2.2)

**Withdrawal**															
All-cause	30	5562	8	12	**23 (17 to 37)**	5	1334	13	19	**15 (8.8 to 46)**	5	2061	20	32	**7.7 (6.0 to 11)**
Lack of efficacy	30	5463	1.2	4.4	**25 (21 to 34)**	6	1435	3.3	7.5	**24 (14 to 69)**	6	2320	4.0	12	**11 (9.0 to 16)**
Adverse event	31	5787	1.6	0.6	***120 (67 to 560)***	7	1657	3.4	1.5	***52 (29 to 260)***	7	2868	3.3	1.8	***65 (37 to 250)***

**Adverse events**															
All cause	18	2862	50	30	***4.9 (4.2 to 6.0)***	3	590	47	25	***4.6 (3.4 to 7.2)***	insufficient data				
Serious	17	2591	2.5	2.4	not calculated	7	1655	1.2	1.1	not calculated	5	1984	2.2	3.2	not calculated
Headache	34	6386	17	5.2	***8.6 (7.6 to 10)***	7	1810	13	3.4	***11 (8.5 to 14)***	6	2411	15	4.1	***9.6 (7.9 to 12)***
Dyspepsia	26	4967	7.8	2.3	***18 (15 to 23)***	6	1401	10	0.2	***11 (8.8 to 14)***	5	1972	3.8	0.3	***31 (22 to 48)***
Flushing	33	6363	13	1.9	***9.0 (8.1 to 10)***	6	1530	4.8	0.2	***24 (18 to 38)***	5	1984	13	0.8	***8.0 (6.9 to 9.6)***
Rhinitis	21	4283	5.4	2.1	***31 (23 to 47)***	2	712	3.1	0.5	not calculated	5	2212	7.9	3.6	***23 (16 to 42)***

NNT is given in standard font, **NNTp in bold**, and ***NNH in bold italic***. No NNT/NNTp/NNH was calculated unless there was a statistically significant difference

There was much less consistency for information on withdrawals. For instance, all cause and lack of efficacy withdrawals were considerably higher with placebo in vardenafil trials (32% and 12%) than with sildenafil trials (12% and 4.4%), with tadalafil intermediate between them. A similar gradient occurred for all cause (Figure 2) and lack of efficacy withdrawals with PDE-5 inhibitors, resulting in lower (better) NNTp values with vardenafil and tadalafil than sildenafil because of these higher absolute rates. Adverse event withdrawals were actually lower with sildenafil than with tadalafil or vardenafil.

Event rates for particular adverse events tended to be consistent between the PDE-5 inhibitor studies, with minor differences. About half the men reported at least one adverse event, though serious adverse events never occurred more frequently with PDE-5 inhibitor than with placebo. Headache was consistently the most commonly reported individual adverse event. Flushing, dyspepsia, or rhinitis/nasal congestion were also common, though with different rates occurring with different PDE-5 inhibitors.

### Other adverse events

Other adverse events were reported inconsistently. Back pain and myalgia or increased CPK levels were mentioned consistently in tadalafil studies, but neither of the other PDE-5 inhibitors (Table 3). Other adverse events ('flu syndrome, limb pain, CV events, fatigue, priapism, and nausea) were reported on in only a small minority of men in trials. Whether visual disturbances occurred was recorded for a large proportion of men, but not in consistent terms, limiting the ability to pool data. Most studies reported that visual disturbances were uncommon.

### Relationship between dosing and intercourse

There was generally no information on intervals between dosing and timing of intercourse, either for speed of onset of effect or duration of effect. Table 10 shows the only consistent information concerning timing of intercourse, from four studies [58,59,61,63] comparing tadalafil 20 mg with placebo. The majority of attempts and successful attempts occurred within four hours, and about 90% within 12 hours. Success rates with tadalafil 20 mg and placebo did not differ, whatever time intercourse occurred. No other studies provided useful information relating to timing of intercourse after dosing.

**Table 10:** Relationship of dosing to time and success of intercourse for tadalafil 20 mg and placebo in four trials

	**Time after dose (hours)**			
	
**Outcome**	**<0.5 to 4**	**>4 to 12**	**>12 to 24**	**>24 to 36**
**Percent of total attempts at intercourse**				
Placebo	75	15	9.0	1.7
Tadalafil 20 mg	61	23	12	3.7

**Percent of successful attempts at intercourse**				
Placebo	74	15	8.4	3.3
Tadalafil 20 mg	60	23	13	4

**Percent of attempts that were successful**				
Placebo	26	26	24	50
Tadalafil 20 mg	65	68	72	74

## Discussion

This analysis comprised 50 randomised comparisons of PDE-5 inhibitors with placebo, in more than 12,000 men. Trials included were all randomised and double blind, and almost all (47/50) were of sufficiently high quality (score of 3 or more out of 5) to avoid major known sources of bias [73].

There was no direct comparison between one PDE-5 inhibitor and another that satisfied our inclusion criteria. Three studies did make a direct comparison between sildenafil and tadalafil, with no placebo. They are not included in the analysis because one [74] was open, another [75] was not convincingly double-blind, and the third [76] used only four tablets per patient. All three studies looked at duration of effect, and two looked at patient preferences. In these less than adequate trials, both drugs seem to be equally effective up to 12 hours after dosing, and both drugs were well tolerated.

Published reports of three PDE-5 inhibitors for treatment of male erectile dysfunction, while largely similar, had interesting differences. These were principally in the underlying aetiology of erectile dysfunction, regions of the world where studies were performed, and in exclusion criteria used to select men for the individual trials. Sildenafil was studied in 10 different clinical conditions, compared with only three for tadalafil and vardenafil (Table 1). The only condition missing for sildenafil was after prostatectomy, where studies could not be included because they were not randomised. Most studies were performed in North America and Europe (Table 2), though sildenafil was studied in men in every inhabited continent, with 27% of men studied being in Asia, South America, or Africa.

Perhaps the most obvious difference between trials of different drugs was the use of different exclusion criteria in individual studies. Five of eight tadalafil studies, and six of seven vardenafil studies excluded men previously unresponsive to PDE-5 inhibitors, thus permitting enrolment to be enriched by responders compared with sildenafil studies, in which such an exclusion would not have been used because it was the first available PDE-5 inhibitor. It is not clear how this major difference might have affected the measured performance of the drugs. Exclusion of non-responders to other PDE-5 inhibitors might be expected to enhance the measured performance of any other PDE-5 inhibitor under test, making it look better in indirect comparisons. However, one vardenafil study including men previously unresponsive to sildenafil [70] was not greatly different from those overall.

The other major difference was in the reporting of outcomes of studies, which varied greatly between the three PDE-5 inhibitors. Common outcomes were responses to a global question about improved erections ("Has the treatment you have been taking over the past four weeks improved your erections?"), and scores and change in score for the erectile function domain. Some outcomes were frequently reported in trials of one treatment, but not others. For example, final scores and changes from baseline for IIEF questions 3 and 4, were reported for most sildenafil, but not tadalafil or vardenafil trials.

There were clear differences in the philosophy of reporting of efficacy and harm, principally between the first PDE-5 inhibitor sildenafil, and the subsequent ones, tadalafil and vardenafil (Table 3). We could find no explanation for this, nor any philosophical discussion about the clinical and practical importance of different outcomes. Few studies provided an estimate of how many men had an outcome approximating erections sufficiently rigid for penetration followed by successful intercourse. This simple pragmatic outcome is more relevant to affected men, their partners, and their professional advisers than an average movement on a scale like IIEF, which itself comprises a number of different questions. Useful outcomes, like the number of men in whom the proportion of successful attempts at sexual intercourse was more than 40%, or 60% or more, known to be recorded in clinical trial reports for sildenafil [6], were almost never reported in published papers. Incomplete reporting of efficacy outcomes has been reported before, for chronic [4] and acute [77] pain.

Withdrawal from studies for any cause, because of lack of effect, or because of adverse events was commonly reported, while numbers of men with at least one adverse event and occurrence of severe adverse events, was recorded for a minority of trials. Serious adverse events were recorded more frequently in tadalafil and vardenafil trials than sildenafil trials, perhaps reflecting recency of studies. The use of a cut-off level for reporting individual adverse events limited the available information for these outcomes.

Analysis of the individual PDE-5 inhibitors (Tables 4, 5, 6, 7) showed that the two top doses (including dose-optimisation schedules) had very similar efficacy for all three interventions. This consistency justified pooling information from the two top doses.

Using this strategy to compare the three PDE-5 inhibitors demonstrated remarkable consistency between them on the basis of available data for any commonly reported outcome. Absolute rates for placebo varied little, showing no major difference between patients studied. The only exception to this was for withdrawals, where tadalafil and vardenafil studies had higher withdrawal rates with placebo and PDE-5 inhibitor than did sildenafil. There was no obvious reason for this. It was perhaps surprising that sildenafil compared well with tadalafil and vardenafil given the much greater number of conditions studied, the wider geographical spread, and that tadalafil and vardenafil studies used a form of enriched enrolment. Overall, sildenafil offered most information, and a trend towards better efficacy and lower adverse events.

How useful is this method of indirect comparison of equivalent doses of PDE-5 inhibitors? It is clearly superior to examining the little inadequate information on direct comparison, and to any superficial examination of individual trials, subject, as each will be, to the random play of chance [13]. Large collections of data from high quality, valid, trials are less subject to the vagaries of chance than smaller individual studies. Large direct comparisons may be better, but the evidence is that when large amounts of trial data exist, direct comparisons give no different result from indirect comparisons [1]. Further investigation of differences between comparable doses would require access to detailed clinical trial reports to report outcomes available but not published.

More important than direct or indirect comparison of different PDE-5 inhibitors is the question of utility of outcomes. With over 12,000 men in clinical trials over a decade, it is surprising that we have no consensus of what is a useful outcome, and how a useful outcome is reported. Individual patient analysis of clinical trial data illustrates how standard trials could be better reported [6,77], and this has already been done for PDE-5 inhibitors [6].

## Competing interests

RAM & HJM have received lecture fees from pharmaceutical companies. All authors have received research support from charities and government sources at various times. This work was supported by an unrestricted educational grant from Pfizer Ltd. The terms of the financial support from Pfizer included freedom for authors to reach their own conclusions, and an absolute right to publish the results of their research, irrespective of any conclusions reached. Pfizer did have the right to view the final manuscript before publication, and did so. No author has any direct stock holding in any pharmaceutical company.

## Authors' contributions

RAM was involved with planning the study, data extraction, analysis, and preparing a manuscript; SD with data extraction, analysis, and writing; HJM with planning, analysis and writing. All authors read and approved the final manuscript.

## Pre-publication history

The pre-publication history for this paper can be accessed here:



## Supplementary Material

Additional File 1 General trial details of PDE-5 inhibitors Included studies, with clinical conditions, country, treatment, dose, duration and quality score Click here for file

Additional File 2 Efficacy outcomes, withdrawals and adverse events Included studies, with efficacy outcomes, withdrawals, and adverse events (patients with any adverse event, and severe, serious and treatment-related adverse events) Click here for file

Additional File 3 Individual adverse events Included studies, details of particular adverse events Click here for file
