# Supplementary material for: Indirect comparison of interventions using published randomised trials: systematic review of PDE-5 inhibitors for erectile dysfunction
Source: BMC Urol. 2005 Dec 14;5:18. doi: 10.1186/1471-2490-5-18 (PMC1343572; doi:10.1186/1471-2490-5-18)
Supplement: Additional File 1 — General trial details of PDE-5 inhibitors Included studies, with clinical conditions, country, treatment, dose, duration and quality score [file 1471-2490-5-18-S1.pdf]

Additional file 1: General trial details of PDE-5 inhibitors

Sildenafil

Reference	Patients	Country	Treatment	Duration	QS
Boolell et al. 1996	History of ED >6 months (mean 3.4 years)<br>ED severity: no details<br>Age:48 (36-63) years	UK	(1) sildenafil 25 mg<br>(2) placebo<br>x-over n=12<br>Daily, 1-2 hours before likely intercourse	2x1 week	R1, D1, W1
Padma-Nathan et al. 1998 [103]<br>See also: Goldstein et al. 1998 dose-escalation study	History of ED >6 months (mean 5 yrs)<br>ED severity: no details<br>Age:60 (26-81) years<br>Organic 59%, Psychogenic 15%<br>Mixed 26%	USA	(1) sildenafil 25-100 mg, n=163<br>(2) placebo, n=166<br>As needed, approx one hour before intercourse, max one dose/day	12 weeks	R1, D2, W1
Goldstein et al. 1998 dose-response study<br><br>[102]	History of ED >6 months (mean 3 years)<br>ED severity: no details<br>Age:58 (20-87) years<br>Organic 78%, Psychogenic 9%<br>Mixed 13%	USA	(1) sildenafil 25 mg, n=102<br>(2) sildenafil 50 mg, n=107<br>(3) sildenafil 100 mg, n=107<br>(4) placebo, n=216<br>As needed, approx one hour before intercourse, max one dose/day	24 weeks	R1, D2, W1
Derry et al. 1998, See also: Maytom et al. 1999<br><br>[358]	History of ED due to spinal cord injury (T6-L5) >6 months (mean 7 years), with grade 2 or more reflexogenic response to PVS<br>ED severity: no details<br>SC lesion: classified<br>Age:33 (21-49) years	UK	(1) sildenafil 50 mg, n=12<br>(2) placebo, n=13<br>As needed, approx one hour before intercourse, max one dose/day	4 weeks	R1, D2, W1
Giuliano et al. 1999<br><br>[367]	ED due to spinal cord injury >6 months (mean 10 years)<br>ED severity: no details<br>SC lesion: classified<br>Age:38 (19-63) years	Europe, Australia	(1) sildenafil 25-100 mg, n=175<br>(2) placebo, n=174<br>{n=completers; 178 randomised}<br>As needed, approx one hour before intercourse, max one dose/day	2x6 week x-over with 2 week washout	R2, D2, W1
Dinsmore et al. 1999<br><br>[359]	History of ED >6 months (most >3 years)<br>ED severity: no details<br>Age:56 (29-89) years<br>Organic 20%, Psychogenic 40%, Mixed 38%, Other 2%	UK	(1) sildenafil 25-100 mg, n=57<br>(2) placebo, n=54<br>As needed, approx one hour before intercourse, max one dose/day	12 weeks	R2, D2, W1
Montorsi et al. 1999<br><br>[364]	History of ED >6 months (most >4 years)<br>ED severity: no details<br>Age:56 (19-79) years<br>Oragnic 32%, Psychogenic 25%, Mixed 43%	Denmark, Ireland, Italy, Norway, Sweden, UK	(1) sildenafil 25 mg, n=128<br>(2) sildenafil 50 mg, n=132<br>(3) sildenafil 100 mg, n=127<br>(4) placebo, n=127<br>As needed, approx one hour before intercourse, max one dose/day	12 weeks	R2, D2, W1
Rendell et al. 1999<br><br>[104]	History of ED >6 months (mean 5 years) and diabetes (mean 12 years)<br>ED severity: no details<br>Age:57 (27-79) years<br>Organic 95%, Mixed 5%	USA	(1) sildenafil 25-100 mg, n=136<br>(2) placebo, n=132<br>As needed, approx one hour before intercourse, max one dose/day	12 weeks	R2, D2, W1
Tan et al. 2000	History of ED >6 months (most >3 years)<br>ED severity: no details<br>Age:52 (26-78) years<br>Organic 63%. Psychogenic 13%, Mixed 24%	SE Asia (Malaysia, Singapore, Philippines)	(1) sildenafil 25-100 mg, n=127<br>(2) placebo, n=127<br>As needed, approx one hour before intercourse, max one dose/day	12 weeks	R1, D2, W0
Olsson et al. 2000	History of ED >3 months (most >4 years), not organic aetiology<br>ED severity: no details, but had 1 or more g3/4 erection in prev 4 weeks<br>Age: 53 (24-70) years<br>Psychogenic 59%, Mixed 41% (2 pts organic)	UK, France, Sweden	(1) sildenafil 10 mg, n=90<br>(2) sildenafil 25 mg, n=85<br>(3) sildenafil 50 mg, n=81<br>(4) placebo, n=95<br>As needed, approx one hour before intercourse, max one dose/day	4 weeks	R1, D1, W1
Palmer et al. 2000	ED and spina bifida<br>ED severity: no details<br>SC lesions: region given, not classified<br>Age: 19-35 years<br>Neurogenic	USA	(1) sildenafil 25 mg<br>(2) sildenafil 50 mg<br>(3) placebo<br>(4) placebo<br>n=17<br>5 tablets per treatment, complete sets taken in random order<br>As needed, approx one hour before intercourse	4 treatment x-over	R1, D1, W0
Chen et al. 2001	History of ED >6 months (mean 4 years)<br>ED severity: no details<br>Age:60 (26-80) years<br>Organic 82%, Psychogenic 8%, Mixed 10%	Taiwan	(1) sildenafil 25-100 mg, n=119<br>(2) placebo, n=117<br>As needed, approx one hour before intercourse, max one dose/day	12 weeks	R1, D2, W1
Hussein et al. 2001	History of ED (most >4 years)and parkinsonism<br>Age:61 (48-68) years for Parkinson's disease<br>Age:54 (46-61) years for multiple system atrophy		(1) sildenafil 25-100 mg, n=14<br>(2) placebo, n=16<br>As needed, approx one hour before intercourse, max one dose/day	2x10 week x-over	R1, D1, W1
Boulton et al. 2001	History of ED(mean >3 years) and diabetes<br>ED severity: no details<br>Age:59 (38-80) years	Denmark, France, Germany, Sweden, UK	(1) sildenafil 25-100 mg, n=110<br>(2) placebo, n=109	12 weeks	R1, D2, W0
Meuleman et al. 2001<br><br>[363]	History of ED >6 months (most >4 years)<br>ED severity: no details<br>Age:55 (23-82) years<br>Organic 29%, Psychogenic 32%, Mixed 37%, Other 2%	Belgium, France, Germany, Netherlands, UK	(1) sildenafil 25-100 mg, n=159<br>(2) placebo, n=156<br>As needed, approx one hour before intercourse, max one dose/day	26 weeks	R1, D2, W1

Eardley et al. 2001<br><br>[355]	History of ED >6 months (mean 3 years)<br>mild to moderate, with 1 or more gd3/4 erections in previous 4 weeks<br>Age:53 (33-69) years	UK	(1) sildenafil 25-75 mg<br>(2) placebo<br>n=44<br>As needed, 30-60 mins before intercourse, max one dose/day	2x4 week x-over	R1, D2, W1
Lewis et al. 2001	History of ED >6 months (most >3 years)<br>ED severity: no details<br>Age:59 (31-81) years<br>Organic 81%, Psychogenic 4%, Mixed 15%	USA	(1) sildenafil 25-100 mg, n=124<br>(2) placebo, n=123<br>As needed, approx one hour before intercourse, max one dose/day	12 weeks	R1, D2, W1
Seidman et al. 2001	History of ED >6 months (mean >5 years) and depression (HAM-D21 ≥12)<br>ED severity: no details<br>Age:56 ±11 years	USA	(1) sildenafil 25-100 mg, n=74<br>(2) placebo, n=78<br>As needed, approx one hour before intercourse, max one dose/day	12 weeks	R1, D2, W1
Incrocci et al. 2001	ED following radiotherapy for PC<br>ED severity: no details<br>Age:68 (56-79) years	Netherlands	(1) sildenafil 25-100 mg, n=30<br>(2) placebo, n=30<br>As needed, approx one hour before intercourse, max one dose/day	2x6 week x-over	R1, D1, W1
Seibel et al. 2002	ED and chronic renal failure (min 12 haemodialysis hrs/wk)<br>mild to severe (75% mild to mod, 13% severe)<br>Age:48 ±10 years	Brazil	(1) sildenafil 50 mg, n=24<br>(2) placebo, n=24<br>As needed, approx one hour before intercourse, max one dose/day	4 weeks	R2, D2, W1
Becher et al. 2002	History of ED (most >2 years)<br>mild to severe (75% mild to mod/mod, 17% severe)<br>Age: 57 ±11 years	Argentina, Chile, Peru, Uraguay	(1) sildenafil 25-100 mg, n=72<br>(2) placebo, n=71<br>As needed, approx one hour before intercourse, max one dose/day	12 weeks	R1, D1, W1
Young et al. 2002	History of ED >6 months<br>mild to severe (60% mild to mod, 30% severe)<br>Age:54 (2-84) years<br>2 populations: black and Hispanic American<br>Organic 60%, Psychogenic 12%, Mixed 28%	USA	(1) sildenafil 25-100 mg, n=119<br>(2) placebo, n=117<br>As needed, approx one hour before intercourse, max one dose/day	6 weeks<br>1st period	R1, D1, W1
Glina et al. 2002	History of ED >6 months (most >3 years)<br>ED severity: no details<br>Age:57 (27-85) years<br>Brazilian and Mexican<br>Organic 41%, Psychogenic 18%, Mixed 51%	Mexico, Brazil	(1) sildenafil 25-100 mg, n=124<br>(2) placebo, n=121<br>As needed, approx one hour before intercourse, max one dose/day	12 weeks	R1, D2, W1
Gomez et al. 2002	History of ED >6 months (most >3 years)<br>ED severity: no details<br>Age:56 (22-77) years<br>Latin American<br>Organic 59%, Psychogenic 16%, Mixed 25%	Columbia, Ecuador, Venezuela	(1) sildenafil 25-100 mg, n=76<br>(2) placebo, n=82<br>As needed, approx one hour before intercourse, max one dose/day	12 weeks	R1, D2, W1
Lindsey et al. 2002	ED following rectal surgery for cancer and IBD<br>ED severity: 56% severe, 44% mild/mod<br>Age:59 (IQR 49-65)	UK	(1) sildenafil 25-100 mg, n=14<br>(2) placebo, n=18<br>As needed, approx one hour before intercourse, max one dose/day	4 weeks	R2, D2, W1
Nurnberg et al. 2003	ED (for >4 weeks) due to antidepressant treatment for MDD (in remission) (<10 on HAM-D and HAM-A)<br>ED severity: no details<br>Age:45 ±8 years	USA	(1) sildenafil 50-100 mg, n=42<br>(2) placebo, n=41<br>As needed, approx one hour before intercourse, max one dose/day	6 weeks	R2, D2, W1
Padma-Nathan et al. 2003	History of ED >6 months (most >6 years), and previous response to sildenafil<br>ED severity: 40% severe, 21% mild/mod)<br>Age:52 (26-78) years<br>Organic 64%, Psychogenic 4%, Mixed 32%	USA	(1) sildenafil 100 mg, n=115<br>(2) placebo, n=113<br>As needed, approx one hour before intercourse, max one dose/day	4 weeks	R1, D2, W1
Kongkanand et al. 2003<br>Thai arm of ASSESS-2	History of ED >6 months (most <2 years)<br>ED severity: 71% mod or severe, 17% mild/mod)<br>Age:55 (26-77) years<br>Organic 64%, Psychogenic 10%, Mixed 26%	Thailand	(1) sildenafil 25-100 mg, n=63<br>(2) placebo, n=62	12 weeks	R1, D2, W1
Levinson et al. 2003<br>Egypt and S Africa	History of ED >6 months (mean 4 years)<br>ED severity: no details<br>Age:52 (26-76) years<br>Organic 39%, Psychogenic 28%, Mixed 33%	Egypt, S Africa	(1) sildenafil 25-100 mg, n=128<br>(2) placebo, n=126<br>As needed, approx one hour before intercourse, max one dose/day	12 weeks	R1, D2, W1
Stuckey et al. 2003	History of ED >6 months (mean 5 years) and type 1 diabetes<br>ED severity: no details<br>Age:47 (25-69) years<br>Organic 72%, Psychogenic 1%<br>Mixed 27%	Australia, Argentina, Brazil, Canada, Italy, Spain, Thailand, Turkey	(1) sildenafil 25-100 mg, n=95<br>(2) placebo, n=93<br>As needed, approx one hour before intercourse, max one dose/day	12 weeks	R2, D2, W1
Choi et al. 2003<br>ASSESS-K (Korea)	History of ED >6 months (mean 5 years)<br>ED severity: no details<br>Age:51 (28-78) years<br>Organic 43%, Psychogenic 34%, Mixed 23%	Korea	(1) sildenafil 25-100 mg, n=66<br>(2) placebo, n=67<br>As needed, approx one hour before intercourse, max one dose/day	8 weeks	R2, D2, W1
Tignol et al. 2004	History of ED (mean 4 years) with MDD (in remission)<br>ED severity: no details<br>Age:53 ±10 years<br>Organic 5%, Psychogenic 68%, Mixed 27%	Belgium, France, Germany. Italy, Spain	(1) sildenafil 25-100 mg, n=83<br>(2) placebo, n=85<br>As needed, approx one hour before intercourse, max one dose/day	12 weeks	R1, D2, W1
Safarinejad et al. 2004	History of ED >6 months (mean >3 years) and diabetes (mean 11 years)<br>ED severity: no details<br>Age:46 (35-68) years<br>Organic 96%, Psychogenic/Mixed 4%	Iran	(1) sildenafil 100 mg, n=144<br>(2) placebo, n=138<br>As needed, approx one hour before intercourse, max one dose/day	16 weeks	R2, D2, W1

DeBusk et al. 2004	History of ED (mean 5 years) with stable CHD<br>ED severity: no details<br>Age:62 (39-82) years<br>Organic 47%, Psychogenic 5%, Mixed 48%	USA	(1) sildenafil 25-100 mg, n=74<br>(2) placebo, n=76	12 weeks	R2, D1, W1
Mahon et al. 2005	History of ED (0.5-7 years) and on peritoneal dialysis (0.5-5 years).<br>ED severity: no details<br>Age: 53 (26-74) years	UK	(1) sildenafil 50-100 mg, n=13<br>(2) placebo, n=13<br>As needed, approx one hour before intercourse, max one dose/day	2x4 week x-over	R0, D1, W1
Fowler et al. 2005	History of ED >6 months (most >4 years) and MS >1 year (mean 10 years)<br>ED severity: no details<br>Age:46 (23-73) years	USA, Europe	(1) sildenafil 25-100 mg, n=104<br>(2) placebo, n=113	12 weeks	R2, D2, W1

Tadalafil

Reference	Patients	Country	Treatment	Duration	QS
Padma-Nathan et al. 2001	History of ED >3 months<br>ED severity: mild to severe (25% severe, 66% mild to mod/mod)<br>Age:56 ±11 years<br>Excl: prev unsuccessful use of PDE5 inhibitor	?USA<br>>80% Caucasian	(1) tadalafil 2 mg, n=35<br>(2) tadalafil 5 mg, n=37<br>(3) tadalafil 10 mg, n=36<br>(4) tadalafil 25 mg, n=36<br>(5) placebo, n=35<br>As needed, max one dose/day, 14 doses in total	3 weeks	R1, D1, W1
Saenz de Tejada et al. 2002	Diabetes and history of ED >3 months<br>mild to severe<br>Age:56 ±9 years	Spain<br>99.5% white	(1) tadalafil 10 mg, n=73<br>(2) tadalafil 20 mg, n=72<br>(3) placebo, n=71<br>As needed, max one dose/day	12 weeks	R1, D1, W1
Porst et al. 2003	History of ED >3 months<br>ED severity: (severe 35%, mod 25%)<br>Age:57 (22-87) years<br>Organic 35%, Psychogenic 30%, Mixed 35%		(1) tadalafil 20 mg, n=175<br>(2) placebo, n=173<br>2 attempts/4 weeks	2x4 weeks<br>Period 1: attempt 24 hrs after dosing<br>Period 2: attempt 36 hrs after dosing	R2, D2, W1
Eardley et al. 2004	History of ED >3 months (most >12 months)<br>ED severity: mild to severe (severe 40%, mod 255)<br>Age:53 (26-78) years<br>Organic 35%, Psychogenic 29%, Mixed 36%<br>Excl: prev unsuccessful use of sildenafil	?UK, Italy, USA<br>(Western European)	(1) tadalafil 20 mg, n=168<br>(2) placebo, n=52<br>As needed, max one dose/day	12 weeks	R1, D1, W1
Skoumal et al. 2004	History of ED >3 months (most >12 months)<br>ED severity: mild to severe (severe 23%, mod 30%)<br>Age:53 (26-78) years<br>Organic 29%, Psychogenic 16%, Mixed 56%<br>Excl due to prev unsuccessful use of sildenafil at discression of investigators	Czech Republ, Poland, Slovakia, Israel, Hungary, Lebanon, Romania	(1) tadalafil 20 mg, n=301<br>(2) placebo, n=102<br>As needed, max one dose/day	12 weeks	R1, D1, W1
Seftel et al. 2004	History of ED >3 months (most >12 months)<br>ED severity: mild to severe (severe 42%, mod 26%)<br>Age:59 ±10 years<br>Organic 57%, Psychogenic 10%, Mixed 33%<br>Excl: prev unsuccessful use of sildenafil	USA, Puerto Rica<br>>70% white, 12% Hispanic	(1) tadalafil 20 mg, n=159<br>(2) placebo, n=48<br>As needed, max one dose/day	12 weeks	R2, D2, W1
Montorsi et al. 2004	ED following NSRRP (12-48 months before)<br>mild to severe (66% with postop penile tumescence)<br>Age: 60 ±5 years	Canada, Germany, Italy, Netherlands, Spain, UK, USA<br>>90% white	(1) tadalafil 20 mg, n=201<br>(2) placebo, n=102<br>As needed, max one dose/day	12 weeks	R1, D2, W1
Carson et al. 2005	History of ED >3 months (most >12 months)<br>ED severity: mild to severe (severe 50%, mod 17%)<br>Organic 83%, Psychogenic 3%, Mixed 14%<br>Age:59 ±10 years<br>Excl: prev unsuccessful use of sildenafil	USA<br>>80% white, 15% African	(1) tadalafil 20 mg, n=146<br>(2) placebo, n=49<br>As needed, max one dose/day	12 weeks	R1, D1, W0

Vardenafil

Reference	Patients	Country	Treatment	Duration	QS
Porst et al. 2001	History of ED >6 months, most >2 years<br>ED severity: mild to severe (33% severe, 35% moderate)<br>Age:52 years<br>Organic 30%, Psychogenic 27%, Mixed 43%<br>Excl: prev unsuccessful use of PDE5 inhibitor	Belgium, France, Germany, Netherlands, Poland, RSA, USA	(1) vardenafil 5 mg, n=146<br>(2) vardenafil 10 mg, n=140<br>(3) vardenafil 20 mg, n=147<br>(4) placebo, n=147<br>[ITT]<br>As needed, approx one hour before intercourse, max one dose/day	12 weeks	R1, D2, W1
Hellstrom et al. 2002	History of ED >6 months (most >3 years)<br>ED severity: mild to severe (severe 38%, mod 30%)<br>Age:57 years<br>Organic 58%, Psychogenic 7%, Mixed 35%<br>Excl: prev unsuccessful use of PDE5 inhibitor	USA, Canada	(1) vardenafil 5 mg, n=205<br>(2) vardenafil 10 mg, n=206<br>(3) vardenafil 20 mg, n=197<br>(4) placebo, n=197<br>[ITT]<br>As needed, approx one hour before intercourse, max one dose/day	26 weeks	R2, D1, W1

Goldstein et al. 2003	History of ED >6 months (most >3 years), and diabetes<br>ED severity: mild to severe (severe 55%, mod 24%)<br>Age:57 years<br>Organic 82%, Psychogenic <1%, Mixed 18%<br>Excl: prev unsuccessful use of PDE5 inhibitor	USA, Canada	(1) vardenafil 10 mg, n=149<br>(2) vardenafil 20 mg, n=141<br>(3) placebo, n=140<br>[ITT]<br>As needed, approx one hour before intercourse, max one dose/day	12 weeks	R2, D1, W1
Brock et al. 2003	History of ED following NSRRP >6 months,<5 years (surgery mean 1.7 yr ago)<br>ED severity: mild to severe (severe 70%, mod 17%)<br>Age:60 years<br>Excl: prev unsuccessful use of PDE5 inhibitor	USA, Canada	(1) vardenafil 10 mg, n=139<br>(2) vardenafil 20 mg, n=147<br>(3) placebo, n=137<br>[ITT]<br>As needed, approx one hour before intercourse, max one dose/day	12 weeks	R2, D2, W1
Hatzichristou et al. 2004	ED (most >2 years)<br>ED severity: no details<br>Age:54<br>Organic 40%, Psychogenic 20%, Mixed 40%<br>Excl: prev unsuccessful use of PDE5 inhibitor	Austria, France, Germany, Greece, Italy, Nehterlands, Spain, Switzerland, UK	(1) vardenafil 5-20 mg, n=155<br>(2) placebo, n=154<br>[ITT]<br>As needed, approx one hour before intercourse, max one dose/day	12 weeks	R2, D2, W1
Carson et al. 2004	ED (most >4 years),<br>ED severity: moderate to severe (severe 54%, mod 36%), previously unresponsive to sildenafil<br>Age: 60 (23-88) years<br>Organic 62%, Psychogenic 6%, Mixed 32%	Many ?USA, UK, Canada, Greece, Australia, Denmark, Brazil	(1) vardenafil 5-20 mg, n=220<br>(2) placebo, n=225<br>[ITT]<br>As needed, approx one hour before intercourse, max one dose/day	12 weeks	R1, D2, W1
Nagao et al. 2004	History of ED >6 months (most <3 years)<br>ED severity: mild to severe (severe 32%, mod 35%)<br>Organic 65%, Psychogenic 90%, Mixed 5%<br>Age:51 (21-70) years<br>Excl: prev unsuccessful use of PDE5 inhibitor	Japan	(1) vardenafil 5 mg, n=67<br>(2) vardenafil 10 mg, n=75<br>(3) vardenafil 20 mg, n=66<br>(4) placebo, n=71<br>[ITT]<br>As needed, approx one hour before intercourse, >24 hrs between doses	12 weeks	R2, D2, W1
