# Supplementary material for: Indirect comparison of interventions using published randomised trials: systematic review of PDE-5 inhibitors for erectile dysfunction
Source: BMC Urol. 2005 Dec 14;5:18. doi: 10.1186/1471-2490-5-18 (PMC1343572; doi:10.1186/1471-2490-5-18)
Supplement: Additional File 2 — Efficacy outcomes, withdrawals and adverse events Included studies, with efficacy outcomes, withdrawals, and adverse events (patients with any adverse event, and severe, serious and treatment-related adverse events) [file 1471-2490-5-18-S2.pdf]

Additional file 2: Efficacy outcomes, withdrawals and adverse events

Sildenafil trials

Study	Dose and number of patients	Doses taken median (range)	Taking max dose at endpoint (n)	Improved erections GAQ1 (n)	Mean number of erections per week (grade 3/4)	Successful attempts at SI (mean per patient, %) SEP	More than 60% attempts successful (n)	More than 40%	Final score (SD) IIEFQ3
Boolell et al. 1996	placebo, n=12<br>25 mg, n=12	7/wk<br>7	n/a	2/12<br>10/12	1.3 (0.5-2.7)<br>6.1 (3.2-11.4)	no data	no data	no data	no data
Padma-Nathan et al. 1998	placebo, n=166<br>25-100 mg, n=163	29/12 wk<br>32	158/166<br>121/163	23/118<br>101/136	no data	20<br>65	(75%) 21/138<br>81/137	no data	2.3 (0.1)<br>3.9 (0.1)
Goldstein et al. 1998	placebo, n=216<br>25 mg, n=102<br>50 mg, n=107<br>100 mg, n=107	no data	n/a	54/216<br>57/102<br>82/107<br>90/107	0.8<br>1.1<br>1.7<br>1.6	no data	no data	no data	2.2 (0.2)<br>3.2 (0.2)<br>3.5 (0.2)<br>4.0 (0.2)
Derry et al. 1998	placebo, n=13<br>50 mg, n=12	8/4 wk<br>8	n/a	1/13<br>9/12	0.4<br>1.8	15<br>30	no data	no data	no data
Giuliano et al. 1999	placebo, n=174<br>25-100 mg, n=175	7/6 wk<br>10	no data<br>102/175	7/168<br>127/168	no data	0<br>55	6/174<br>73/175	no data	2.2<br>3.8
Dinsmore et al. 1999	placebo, n=54<br>25-100 mg, n=57	1.7/wk<br>2.1	no data<br>79% on 50/100	10/54<br>46/57	0.6<br>1.7	30<br>73	no data	no data	1.7<br>3.6
Montorsi et al. 1999	placebo, n=127<br>25 mg, n=128<br>50 mg, n=132<br>100 mg, n=127	20-30 across groups/12 wks	n/a	27/114<br>80/119<br>95/122<br>101/118	no data	24<br>64<br>73<br>73	no data	no data	2.2<br>3.2<br>3.7<br>3.8
Rendell et al. 1999	placebo, n=132<br>25-100 mg, n=136	25 (2-83)/12 wk<br>31 (3-81)	127/132<br>126/136	13/127<br>74/131	no data	no data	no data	no data	2.0<br>3.2
Tan et al. 2000	placebo, n=127<br>25-100 mg, n=127		no data<br>81/125	40/121<br>109/125	no data	30<br>78	no data	no data	2.6<br>4.2

Sildenafil trials

Mean (SD) change EFQ3	Final score (SD) IIEFQ4	Mean (SD) change EFQ4	Final score (SD) EF Domain	Mean (SD) change EF Domain	Normal EF score (26-30) at endpoint (n)	Withdrawals			Adverse events			
						Total	Lack of Efficacy	Adverse events	All cause	Severe	Serious	Treatment related
no data	no data	no data	no data	no data	no data	0/12	0/12	0/12	5/12	0/12	0/12	no data
						0/12	0/12	0/12	6/12	1/12	0/12	
0.2	1.8 (0.1)	0.2	12.2	0.2	no data	13/166	3/166	1/166	no data	no data	no data	no data
1.9	3.6 (0.1)	2.1	22.1	11		9/163	1/163	1/163				
0.1	2.1 (0.2)	0.4	no data	no data	no data	36/216	11/216	1/216	no data	no data	no data	no data
1.2	3.1 (0.2)	1.7				15/102	3/102	1/102				
1.6	3.5 (0.2)	2.0				8/107	2/107	1/107				
2.0	3.9 (0.2)	2.2				8/107	0/107	2/107				
no data	no data	no data	no data	no data	no data	1/14	0/14	0/14	4/13	no data	1/13	1/13
						0/13	0/13	0/13	5/12		0/12	2/12
0.2	1.7	0.1	no data	no data	no data	4/174	0/174	2/174	no data	no data	no data	1/174
2.9	3.6	2.1				6/175	0/175	5/175				3/175
0.03	1.6	0.08	10.1	-0.2	no data	11/54	1/54	0/54	no data	14 total	no data	no data
1.9	3.7	2.1	21.5	11.2		3/57	1/57	0/57				
-0.03	2.0	0.1	no data	no data	no data	no data	no data	1/127	42/127	no data	7 total	11/127
1.0	3.0	1.2						0/128	63/128	1/128		39/128
1.5	3.4	1.6						1/132	80/132	2/132		50/132
1.6	3.6	1.8						5/127	92/127	5/127		72/127
0.4	1.6	0.2	10.4	1.2	no data	11/132	1/132	1/132	no data	no data	no data	1/132
1.4	2.9	1.4	17.5	7.4		5/136	1/136	1/136				22/136
0.3	2.4	0.5	15.5	2.2	no data	11 total	4/127	0/127	29/127	4 total	1/127	13/127
2.0	4.2	2.2	25.1	11.8			0/127	1/127	42/127		1/127	29/127

Olsson et al. 2000	placebo, n=95<br>10 mg, n=90<br>25 mg, n=85<br>50 mg, n=81	no data	no data	35/91<br>54/84<br>65/82<br>67/76	2.1<br>2.8<br>3.0<br>3.6	no data	no data	no data	2.4<br>2.9<br>3.0<br>3.3
Palmer et al. 2000	placebo, n=17<br>25 mg, n=17<br>50 mg, n=17	5 x 2<br>5<br>5	n/a	2/15<br>12/15	no data	no data	no data	no data	no data
Chen et al. 2001	placebo, n=113<br>25-100 mg, n=111	41/12 wk<br>35	106/113<br>79/111	43/113<br>98/111	no data	30<br>62	no data	no data	3.0<br>4.2
Boulton et al. 2001	placebo, n=109<br>25-100 mg, n=110	10.4 ±6/4 wk<br>10.9 ±6	no data<br>85/110	11/103<br>66/102	no data	14.4 (8-23)<br>58.8 (48-69)	no data	no data	1.9 (0.2)<br>3.4 (0.2)
Meuleman et al. 2001	placebo, n=156<br>25-100 mg, n=159	34 (3-162)/26 wk<br>49 (3-181)	no data<br>78/159	36/159<br>126/159	1.3<br>3.2	no data	no data	no data	2.2 (0.2)<br>3.7 (0.1)
Eardley et al. 2001	placebo, n=44<br>25-100 mg, n=44	2.6 (0.3)/wk<br>3.4 (0.3)	no data	9/36<br>34/36	1.4<br>4.2	no data	no data	no data	no data
Lewis et al. 2001	placebo, n=123<br>25-100 mg, n=124	34/12 wk<br>33	118/123<br>101/124	21/123<br>87/124	no data	no data	no data	no data	2.2<br>3.7
Seidman et al. 2001	placebo, n=78<br>25-100 mg, n=74	3.0/wk<br>3.6	73/75<br>57/72	8/70<br>60/66	no data	no data	no data	no data	2.2 (0.2)<br>3.7 (0.3)
Incrocci et al. 2001	placebo, n=60<br>25-100 mg, n=60	no data	no data<br>54/60	5/60<br>27/60	no data	18<br>55	no data	no data	1.6 (1.1)<br>2.8 (1.7)
Seibel et al. 2002	placebo, n=24<br>50 mg, n=21	no data	n/a	2/21<br>17/20	no data	no data	no data	no data	2.8 (0.8)<br>4.0 (1.1)
Becher et al. 2002	placebo, n=71<br>25-100 mg, n=72	31 (1-83)<br>33 (1-80)	57/69<br>36/71	22/65<br>51/66	no data	26.5<br>62.5	no data	no data	2.7 (0.2)<br>3.8 (0.2)
Young et al. 2002<br>Blacks 1st 6-wk period	placebo, n=122<br>25-100 mg, n=124	no data	no data	41/114<br>93/118	no data	no data	no data	no data	3.1<br>4.1
Young et al. 2002<br>Hisp 1st 6-wk period	placebo, n=98<br>25-100 mg, n=99	no data	no data	26/89<br>73/89	no data	no data	no data	no data	3.0<br>4.0
Glina et al. 2002	placebo, n=121<br>25-100 mg, n=124	32<br>36	no data<br>71/124	no data	no data	no data	no data	no data	2.6 (0.2)<br>3.9 (0.2)

0.4<br>0.9<br>1.0<br>1.3	2.1<br>2.6<br>2.8<br>3.2	0.6<br>1.1<br>1.3<br>1.7	no data	no data	no data	9/95<br>7/90<br>7/85<br>11/81	0/95<br>1/90<br>2/85<br>1/81	4/95<br>2/90<br>4/85<br>7/81	no data	no data	3 total	no data
no data	no data	no data	no data	no data	no data	2/17<br>2/17	0/17<br>0/17	0/17<br>0/17	no data<br>sild doses<br>combined	no data	no data	no data
0.7<br>1.9	2.9<br>4.1	0.9<br>2.1	18.1<br>24.3	4.5<br>11.7	no data	6/117<br>10/119	1/117<br>1/119	1/117<br>1/119	58/117<br>76/119	no data<br>1/119	4/117<br>4/119	22/117<br>52/119
0.1<br>1.7	1.8 (0.2)<br>3.4 (0.2)	0.4<br>2.9	11.5 (1.2)<br>20.4 (1.2)	1.1<br>10.0	no data	no data	no data	2/109<br>2/110	no data	no data	no data	7/109<br>41/110
0.6<br>1.7	2.1 (0.2)<br>3.6 (0.2)	0.5<br>1.7	13.3 (0.7)<br>21.9 (0.7)	1.5<br>10.9	no data	77/156<br>35/159	54/156<br>13/159	1/156<br>5/159	no data	no data	6/156<br>8/159	no data
no data	no data	no data	no data	no data	no data	5 total	0/44<br>0/44	1/44<br>0/44	14/43<br>23/43	no data	0/43<br>2/43	no data
0.5<br>2.0	1.9<br>3.5	0.5<br>2.1	no data	no data	no data	12/123<br>7/124	8/123<br>2/124	0/123<br>2/124	no data	0/123<br>1/124	3/123<br>3/124	20/123<br>52/124
0.6<br>2.1	2.0 (0.2)<br>3.9 (0.3)	0.6<br>2.5	12.4<br>23.4	3.1<br>14.1	no data	no data	no data	no data	no data	5 total	no data	10/78<br>35/74
0.1<br>1.3	1.5 (1.0)<br>2.6 (1.7)	0.2<br>1.3	10.6<br>16.9	0.9<br>7.2	no data	0/60<br>0/60	0/60<br>0/61	0/60<br>0/62	no data	0/60<br>0/60	no data	no data
0.1<br>1.2	2.4 (0.9)<br>3.7 (1.3)	-0.1<br>1.3	15.2<br>23.0	-0.2<br>7.0	0/24<br>7/21	3/24<br>1/21	0/24<br>0/21	2/24<br>0/21	5/24<br>3/21	1/24<br>0/21	2/24<br>0/21	no data
0.4<br>1.5	2.5 (0.2)<br>3.6 (0.2)	0.4<br>1.6	15.9 (0.7)<br>20.5 (0.6)	1.34<br>6.0	no data	6/71<br>7/72	0/71<br>0/72	1/71<br>2/72	21/71<br>43/72	1/21<br>2/43	1/71<br>2/72	no data
0.5<br>1.5	2.9<br>3.9	0.6<br>1.7	17.6<br>23.3	3.5<br>9.2	no data	10/122<br>6/123	no data	no data	no data	no data	no data	4/122<br>22/124
0.6<br>1.6	2.6<br>3.8	0.5<br>1.7	16.4<br>23.2	2.9<br>9.7	no data	9/98<br>13/99	no data	no data	no data	no data	no data	11/97<br>26/98
0.5<br>1.9	2.3 (0.2)<br>3.8 (0.2)	0.6<br>2.1	no data	no data	no data	16/121<br>15/124	3/121<br>3/124	0/121<br>1/124	no data	no data	no data	no data

Gomez et al. 2002	placebo, n=82<br>25-100 mg, n=76	no data	no data	38/82<br>59/72	no data	35<br>65	no data	no data	3.3<br>4.0
Lindsey et al. 2002	placebo, n=18<br>25-100 mg, n=14	no data	no data	3/18<br>11/14	no data	no data	no data	no data	no data
Nurnberg et al. 2003	placebo, n=41<br>25-100 mg, n=43	4.5 (2.1)/wk<br>5.3 (2.2)	30/35<br>32/41	no data	no data	no data	no data	no data	3.1 (1.6)<br>4.4 (1.1)
Padma-Nathan et al. 2003	placebo, n=113<br>100 mg, n=115	no data	n/a	no data	no data	no data	no data	no data	no data
Kongkanand et al. 2003	placebo, n=62<br>25-100 mg, n=63	no data	no data	no data	no data	no data	no data	no data	2.8<br>3.8
Levinson et al. 2003	placebo, n=126<br>25-100 mg, n=128	33/12 wk<br>41	114/126<br>86/128	33/122<br>93/126	no data	28<br>69	no data	no data	2.5<br>3.9
Stuckey et al. 2003	placebo, n=93<br>25-100 mg, n=95	10.6/month<br>11.4	no data<br>68/95	split by severity	no data	38<br>65	no data	no data	2.7 (0.5)<br>3.6 (0.5)
Choi et al. 2003	placebo, n=67<br>25-100 mg, n=66	22/8 wk<br>24.5	65/65<br>43/66	18/65<br>54/66	no data	25.9<br>62.0	no data	no data	2.7<br>4.2
Tignol et al. 2004	placebo, n=85<br>25-100 mg, n=83	21 (1-84)<br>31 (1-83)	72/85<br>42/83	28/85<br>68/83	no data	29<br>74	no data	no data	2.6<br>3.8
Safarinejad et al. 2004	placebo, n=138<br>100 mg, n=144	40 (4-97)/16 wk<br>42 (5-102)	n/a	14/128<br>68/134	no data	no data	no data	no data	2.2 (0.2)<br>2.8 (0.2)
DeBusk et al. 2004	placebo, n=76<br>25-100 mg, n=174	8 (1-40)<br>12 (1-36)	63/76<br>55/74	20/72<br>41/70	no data	25<br>51	no data	no data	2.7<br>3.5
Mahon et al. 2005	placebo, n=13<br>50-100 mg, n=13	no data	step inc<br>13/13<br>[illegible]	4/13<br>10/13	no data	no data	no data	no data	1.7<br>3.4
Fowler et al. 2005	placebo, n=113<br>25-100 mg, n=104	no data	108/113<br>67/104	27/113<br>94/104	no data	no data	no data	no data	2.4 (0.2)<br>4.0 (0.2)

0.9<br>1.6	2.9<br>3.8	0.9<br>1.8	18.4<br>22.1	4.8<br>8.5	no data	10/82<br>12/76	1/82<br>0/76	0/82<br>1/76	27/82<br>39/76	no data	0/82<br>0/76	18/82<br>33/76
no data	no data	no data	10.6<br>23.6	3.3<br>13.1	no data	0/18<br>0/14	0/18<br>0/14	0/18<br>0/14	4/18<br>7/14	0/18<br>1/14	0/18<br>0/14	no data
0<br>1.3	2.7 (1.6)<br>4.2 (1.2)	0.1<br>1.3	17.1 (8.1)<br>27.1 (3.7)	0.8<br>9.3	no data	6/41<br>1/43	5/41<br>0/43	1/41<br>1/43	no data	no data	no T-R	no data
no data	no data	no data	no data	no data	no data	5/115<br>4/113	?0/113<br>0/115	0/113<br>2/115	15/113<br>29/115	no data	no data	no data
0.8<br>1.6	2.5<br>3.6	0.7<br>1.6	15.9<br>21.8	3.0<br>8.9	no data	1/62<br>0/63	0/62<br>0/63	0/62<br>0/63	21/62<br>23/63	no data	no data<br>0/63	7/62<br>19/63
0.4<br>1.8	2.2<br>3.7	0.5<br>2.0	no data	no data	no data	11/126<br>15/128	4/126<br>0/128	0/126<br>3/128	43/126<br>63/128	no data	0/126<br>3/128	20/126<br>50/128
0.5<br>1.0	2.2 (0.5)<br>3.3 (0.5)	0.5<br>1.4	14.5<br>20	2.5<br>6	no data	16/93<br>10/95	3/93<br>2/95	3/93<br>2/95	13/93<br>34/95	1/93<br>5/95	1/93<br>0/95	no data
0.1<br>1.6	2.1<br>3.7	0.3<br>2.0	14.5<br>23.4	1.5<br>10.4	no data	4/67<br>0/66	1/67<br>0/66	0/67<br>0/66	25/67<br>46/66	0/67<br>1/66	2/67<br>0/66	14/67<br>37/66
no data	2.1<br>3.5	no data	14<br>24	no data	no data	10/85<br>9/83	4/85<br>1/83	0/85<br>3/83	14/85<br>34/83	no data	no data	no data
0.4<br>1.0	2.0 (0.2)<br>2.9 (0.2)	0.3<br>1.2	11.4<br>16.8	1.3<br>6.3	no data	10/138<br>10/144	3/138<br>1/144	no data<br>8/144	no data	no data	no data	2/138<br>32/144
0.8<br>1.6	2.3<br>3.3	0.7<br>1.7	15.0<br>19.8	4.5<br>9.3	no data	15/76<br>10/74	4/76<br>0/74	2/76<br>3/74	24/76<br>35/74	no data	2/76<br>1/74	no data
no data	1.6<br>3.2	no data	12<br>20	5.3<br>13.3	no data	0/13<br>0/13	0/13<br>0/13	0/13<br>0/13	0/13<br>1/13	0/13<br>0/13	0/13<br>0/13	0/13<br>1/13
0.5<br>2.1	2.0 (0.2)<br>3.9 (0.2)	0.5<br>2.4	no data	no data	no data	25/113<br>2/104	24/113<br>0/104	1/113<br>0/104	49/113<br>68/104	4/113<br>10/104	3/113<br>3/104	7/113<br>44/104

Tadalafil trials

Study	Dose and number of patients	Doses taken median (range)	Taking max dose at endpoint (n)	Improved erections GAQ1 (n)	Mean number of erections per week (grade 3/4)	Successful attempts at SI (mean per patient, %) SEP	More than 60% attempts successful (n)	More than 40%	Final score (SD) IIEFQ3
Padma-Nathan et al. 2001	placebo, n=35<br>2 mg, n=35<br>5 mg, n=37<br>10 mg, n=36<br>25 mg, n=36	max 14	n/a	6/35<br>18/35<br>22/37<br>29/36<br>29/36	no data	26.6<br>45.7<br>61.7<br>69.8<br>70.2	no data	no data	2.5 (0.3)<br>3.5 (0.3)<br>4.2 (0.2)<br>4.1 (0.2)<br>4.2 (0.2)
Saenz de Tejada et al. 2002	placebo, n=71<br>10 mg, n=73<br>20 mg, n=72	no data	n/a	18/71<br>41/73<br>46/72	no data	no data	no data	no data	
Porst et al. 2003	placebo, n=173<br>20 mg, n=175	2+2<br>2+2	n/a	no data	no data	no data	no data	no data	no data
Eardley et al. 2004	placebo, n=52<br>20 mg, n=168	no data	n/a	12/52<br>138/168	no data	29.9<br>73.9	no data	no data	2.5 (1.7)<br>4.3 (1.4)
Skoumal et al. 2004	placebo, n=102<br>20 mg, n=301	2.9/wk<br>3.9/wk	n/a	34/102<br>259/301	no data	34<br>73	no data	no data	no data
Seftel et al. 2004	placebo, n=48<br>20 mg, n=159	no data	n/a	9/48<br>125/159	no data	22.6<br>64.2	no data	no data	no data
Montorsi et al. 2004	placebo, n=102<br>20 mg, n=201	2.1±1.4<br>2.3±1.5	n/a	23/102<br>124/201	no data	19.4<br>40.5	no data	no data	no data
Carson et al. 2005	placebo, n=49<br>20 mg, n=146	no data	n/a	7/49<br>107/146	no data	25.0<br>50.3	no data	no data	2.4<br>3.3

Tadalafil trials

						Withdrawals			Adverse events			
**Mean (SD) change EFQ3**	**Final score (SD) IIEFQ4**	**Mean (SD) change EFQ4**	**Final score (SD) EF Domain**	**Mean (SD) change EF Domain**	**Normal EF score (26-30) at endpoint (n)**	**Total**	**Lack of Efficacy**	**Adverse events**	**All cause**	**Severe**	**Serious**	**Treatment related**
-0.3 (0.2)<br>0.6 (0.2)<br>1.2 (0.2)<br>1.0 (0.2)<br>1.3 (0.2)	2.4 (0.3)<br>3.1 (0.3)<br>3.7 (0.2)<br>4.0 (0.2)<br>4.0 (0.2)	0.2 (0.2)<br>0.8 (0.2)<br>1.4 (0.2)<br>1.7 (0.2)<br>1.7 (0.2)	14.7 (1.2)<br>19.3 (1.5)<br>22.9 (1.0)<br>23.6 (1.1)<br>24.2 (1.2)	1.0 (0.9)<br>4.1 (1.1)<br>7.3 (1.0)<br>7.8 (1.2)<br>9.4 (1.2)	no data	7 total	0/35<br>0/35<br>0/37<br>0/36<br>0/36	0/35<br>0/35<br>0/37<br>2/36<br>0/36	3/35<br>6/35<br>no data<br>no data<br>13/36	no data	0/35<br>1/35<br>0/37<br>0/36<br>1/36	3/35<br>6/35<br>no data<br>no data<br>13/36
no data		no data		0.1<br>6.4<br>7.3	no data	25 total	0/71<br>3/73<br>2/72	1/71<br>1/73<br>4/72	22/71<br>29/73<br>32/72	no data	1/71<br>0/73<br>1/72	no data
no data	no data	no data	no data	no data	no data	no data	no data	no data	no data	no data	no data	no data
0.1 (1.4)<br>1.9 (1.7)	2.1 (1.5)<br>4.1 (1.5)	0.1 (1.3)<br>2.2 (1.7)	13.8 (8.5)<br>24.5 (7.5)	0.4 (6.6)<br>11.1 (27.2)	9/52<br>114/168	9/52<br>16/168	no data	1/52<br>5/168	no data	no data	1/52<br>3/168	no data
no data	no data	no data	17.4 (15.9-18.9)<br>25.1 (24.5-25.8)	1.4 (0.8-2.1)<br>9.8 (9.0-10.6)	16/97<br>188/292	8/104<br>15/305	5/104<br>0/305	0/104<br>1/305	no data	no data	0/104<br>2/305	no data
no data	no data	no data	13.6<br>22.5	0.3 (0.9)<br>9.3 (0.6)	4/48<br>80/159	8/48<br>23/159	5/48<br>3/159	1/48<br>8/159	no data	no data	0/48<br>2/159	no data
no data	no data	no data	13.3<br>17.7	1.1 (0.6)<br>5.3 (0.5)	5/102<br>48/201	26/102<br>40/201	10/102<br>16/201	2/102<br>11/201	27/102<br>104/201	3/102<br>10/201	0/102<br>0/201	no data
0.1 (0.27)<br>1.1 (0.15)	2.0<br>3.1	0 (0.22)<br>1.3 (0.15)	13.5<br>19.5	-0.2 (1.08)<br>6.9 (0.75)	no data	18/49<br>30/146	9/49<br>8/146	1/49<br>8/146	no data	no data	1/49<br>3/146	no data

Vardenafil trials

Study	Dose and number of patients	Doses taken median (range)	Taking max dose at endpoint (n)	Improved erections GAQ1 (n)	Mean number of erections per week (grade 3/4)	Successful attempts at SI (mean per patient, %) SEP	More than 60% attempts successful (n)	More than 40%	Final score (SD) IIEFQ3
Porst et al. 2001	placebo, n=147	26/12 wks	n/a	44/147	no data	39.5	40/147	no data	2.7 (1.5)
	5 mg, n=146	29		96/146		71.1	90/146		3.7 (1.5)
	10 mg, n=140	29		106/140		70.9	80/140		3.9 (1.5)
	20 mg, n=147	31		118/147		74.6	96/147		4.0 (1.4)
Hellstrom et al. 2002	placebo, n=197	no data	n/a	45/197	no data	32.7	no data	no data	no data
	5 mg, n=205			115/205		51.7			
	10 mg, n=206			158/206		64.7			
	20 mg, n=197			159/197		66.7			
Goldstein et al. 2003	placebo, n=140	1.8/wk	n/a	17/140	no data	23	split by baseline severity	no data	no data
	10 mg, n=149	2.1		78/149		49			
	20 mg, n=141	2.0		94/141		54			
Brock et al. 2003	placebo, n=137	no data	n/a	12/137	no data	10	no data	no data	no data
	10 mg, n=139			78/139		37			
	20 mg, n=147			87/147		34			
Hatzichristou et al. 200	placebo, n=154	20/12 wks	140/155	55/154	no data	30	no data	no data	no data
	5-20 mg, n=155	30	110/154	133/155		69			
Carson et al. 2004	placebo, n=221	8.4-9.2/4 wks	no data	32/221	no data	19.9	no data	no data	no data
	5-20 mg, n=227	10.1-11.5	189/227	140/227		50.8			
Nagao et al. 2004	placebo, n=71	no data	n/a	25/71	no data	33.4	no data	no data	3.2
	5 mg, n=67			49/67		63.5			4.0
	10 mg, n=75			64/75		78.5			4.5
	20 mg, n=66			57/66		79.3			4.6

Vardenafil trials

						Withdrawals			Adverse events			
Mean (SD) change EFQ3	Final score (SD) IIEFQ4	Mean (SD) change EFQ4	Final score (SD) EF Domain	Mean (SD) change EF Domain	Normal EF score (26-30) at endpoint (n)	Total	Lack of Efficacy	Adverse events	All cause	Severe	Serious	Treatment related
0.2 (1.5)	2.5 (1.5)	0.5 (1.7)	15.6 (7.3)	1.6	no data	no data	no data	2/152	no data	no data	4/152	no data
1.2 (1.7)	3.5 (1.5)	1.4 (1.7)	20.9 (7.3)	5.7				7/147			4/147	
1.3 (1.5)	3.6 (1.5)	1.5 (1.6)	22.1 (7.5)	8.0				2/141			1/141	
1.5 (1.7)	3.8 (1.4)	1.7 (1.6)	22.8 (7.5)	9.0				1/150			2/150	
no data	no data	no data	14.8	1.2	split by baseline	106/197	39/177	4/182	no data	no data	9/182	13/182
			17.8	5.3	severity	77/205	26/190	8/193			10/193	37/193
			21.2	7.8		55/206	10/196	7/199			6/199	66/199
			21.8	9.0		59/197	9/186	15/188			8/188	79/188
no data	no data	no data	12.6	no data	no data	no data	5/140	2/143	no data	no data	4/143	no data
			17.1				3/149	4/152			3/152	
			19.0				0/141	5/144			4/144	
no data	no data	no data	9.2	0.1	no data	48/145	27/137	1/140	no data	3/140	no data	no data
			15.3	6.0		32/146	8/139	5/140		7/140	3/140	
			15.3	6.1		30/149	14/147	5/147		5/147	no data	
no data	no data	no data	14.5	no data	no data	49/166	20/154	3/164	44/164	no data	5/164	8/164
			23.3			25/167	2/155	5/157	71/157		4/157	45/157
no data	no data	no data	10.5	0.8	12/205	42/233	17/226	3/226	no data	6/226	no data	no data
			17.6	8.3	67/212	26/230	5/231	5/231		5/231		
0.6	2.3	0.8	16.7	3.1	no data	19/72	5/71	4/71	37/71	no severe	no SAEs	15/71
1.2	3.5	1.9	22.4	8.3		9/68	1/67	2/67	39/67	AEs		24/67
1.7	4.2	2.6	25.6	11.8		11/76	0/75	2/75	47/75			34/75
2.1	4.3	2.8	26.0	12.6		6/67	2/66	3/66	49/66			36/66
