# Supplementary material for: Indirect comparison of interventions using published randomised trials: systematic review of PDE-5 inhibitors for erectile dysfunction
Source: BMC Urol. 2005 Dec 14;5:18. doi: 10.1186/1471-2490-5-18 (PMC1343572; doi:10.1186/1471-2490-5-18)
Supplement: Additional File 3 — Individual adverse events Included studies, details of particular adverse events [file 1471-2490-5-18-S3.pdf]

Additional file 3: Individual adverse events

Sildenafil trials

Study	Ascertainment and classification	Reporting cut-off level	Severity	Headache	Dyspepsia	Back pain	Myalgia	Flu syndrome	Flushing	Visual disturbances	CV events	Limb pain	Nasal congestion/ Rhinitis	Fatigue	Priapism
Boolell et al. 1996	Recorded	n/a		no numerical data provided											
Padma-Nathan et al. 1998	Recorded and monitored	5%	most mild to mod, transient	placebo 6/166<br>Sild25-100 30/163	placebo 4/166<br>Sild25-100 9/163				placebo 1/166<br>Sild25-100<br>30/163	placebo 1/166<br>Sild25-100<br>4/163					none
Goldstein et al. 1998	Recorded	5%	usually mild, transient	placebo 14/216<br>Sild25 14/102<br>Sild50 23/107<br>Sild100 32/107	placebo 3/216<br>Sild25 3/102<br>Sild50 12/107<br>Sild100 17/107				placebo 3/216<br>Sild25 13/102<br>Sild50 29/107<br>Sild100 21/107	placebo 1/216<br>Sild25 2/102<br>Sild50 6/107<br>Sild100 10/107			placebo 4/216<br>Sild25 1/102<br>Sild50 3/107<br>Sild100 12/107		none
Maytom et al. 1999	Recorded	none	most mild	placebo 1/13<br>Sild50 0/12	placebo 0/13<br>Sild50 1/12			placebo 1/13<br>Sild50 0/12							
Giuliano et al. 1999	Recorded	2%	most mild or mod	placebo 8/174<br>Sild25-100 30/175	placebo 0/174<br>Sild25-100 5/175				placebo 2/174<br>Sild25-100<br>12/175	placebo 0/174<br>Sild25-100<br>4/175			placebo 0/174<br>Sild25-100<br>3/175		none
Dinsmore et al. 1999	Observed and reported	most common	most mild or mod	placebo 0/64<br>Sild25-100 7/57	placebo 1/54<br>Sild25-100 4/57				placebo 2/54<br>Sild25-100 5/57	placebo 0/54<br>Sild25-100 2/57					
Montorsi et al. 1999	Observed and reported	5%	most mild to mod, transient	placebo 5/127<br>Sild25 26/128<br>Sild50 23/132<br>Sild100 27/127	placebo 2/127<br>Sild25 2/128<br>Sild50 7/132<br>Sild100 14/127	placebo 2/127<br>Sild25 3/128<br>Sild50 8/132<br>Sild100 0/127			placebo 3/127<br>Sild25 16/128<br>Sild50 25/132<br>Sild100 25/127	placebo 2/127<br>Sild25 0/128<br>Sild50 1/132<br>Sild100 14/127					
Rendell et al. 1999	Recorded	3%	most mild to mod, transient	placebo 2/132<br>Sild25-100 15/136	placebo 0/132<br>Sild25-100<br>12/136				placebo 0/132<br>Sild25-100 6/136	placebo 1/132<br>Sild25-100<br>5/136	placebo 6/`132<br>Sild25-100<br>4/136		placebo 2/132<br>Sild25-100<br>13/136 ?		none
Tan et al. 2000	Observed and reported	at least 2 patients	most mild	placebo 10/127<br>Sild25-100 14/127	placebo 1/127<br>Sild25-100 2/127	placebo 3/127<br>Sild25-100<br>2/127	placebo 0/127<br>Sild25-100<br>4/127	placebo 3/127<br>Sild25-100<br>2/127	placebo 6/127<br>Sild25-100<br>10/127	placebo 0/127<br>Sild25-100<br>4/127	placebo 0/127<br>Sild25-100<br>3/127		placebo 5/127<br>Sild25-100<br>7/127		
Olsson et al. 2000	Recorded	5%	most mild or mod	placebo 7/95<br>Sild10 12/90<br>Sild25 20/85<br>Sild50 16/81	placebo 3/95<br>Sild10 4/90<br>Sild25 9/85<br>Sild50 5/81		placebo 1/95<br>Sild10 1/90<br>Sild25 3/85<br>Sild50 6/81	placebo 2/95<br>Sild10 0/90<br>Sild25 5/85<br>Sild50 2/81	placebo 0/95<br>Sild10 4/90<br>Sild25 7/85<br>Sild50 7/81	none		[Arthralgia]<br>placebo 1/95<br>Sild10 1/90<br>Sild25 6/85<br>Sild50 0/81			
Palmer et al. 2000				no data for placebo<br>sild doses combined											
Chen et al. 2001	Observed and reported COSTART	at least 3 patients	most mild	placebo 4/117<br>Sild25-100 8/119	[digest syst]<br>placebo 14/117<br>Sild25-100<br>15/119		placebo 0/117<br>Sild25-100<br>3/119	[resp tract inf]<br>placebo 16/117<br>Sild25-100<br>14/119	placebo 11/117<br>Sild25-100<br>30/119	placebo 1/117<br>Sild25-100<br>3/119	[Palpitation]<br>placebo 2/117<br>Sild25-100<br>4/119	[Arthralgia]<br>placebo 2/117<br>Sild25-100<br>3/119			
Boulton et al. 2001		5%	all mild to mod, transient	Only treatment-related AEs reported											
Meuleman et al. 2001	Observed and reported	most common	most mild to mod, transient	placebo 11/156<br>Sild25-100 15/159	placebo 5/156<br>Sild25-100<br>11/159			placebo 10/156<br>Sild25-100<br>11/159	placebo 0/156<br>Sild25-100<br>18/159	placebo 0/156<br>Sild25-100<br>2/159			placebo 6/156<br>Sild25-100<br>9/159		
Eardley et al. 2001	Observed and volunteered		most mild or mod	placebo 0/43<br>Sild25-100 11/43	placebo 1/43<br>Sild25-100 2/43				placebo 0/43<br>Sild25-100 4/43			[Arthralgia]<br>placebo 2/43<br>Sild25-100 3/43			
Lewis et al. 2001	Recorded	most common	most mild or mod	placebo 1/123<br>Sild25-100 14/124	placebo 0/123<br>Sild25-100 5/124				placebo 1/123<br>Sild25-100<br>17/124	placebo 0/123<br>Sild25-100<br>3/124					

Seidman et al. 2001	Observed and reported	5%	most mild to mod, transient	placebo 5/78<br>Sild25-100 15/74	placebo 0/78<br>Sild25-100 11/74				placebo 1/78<br>Sild25-100 11/74	placebo 1/78<br>Sild25-100 6/74				
Incrocci et al. 2001			all mild to mod, transient	placebo 15/60<br>Sild25-100 42/60	placebo 8/60<br>Sild25-100 32/60		placebo 13/60<br>Sild25-100<br>15/60		placebo 2/60<br>Sild25-100 13/60	placebo 8/60<br>Sild25-100<br>17/60		placebo 12/60<br>Sild25-100<br>22/60		
Seibel et al. 2002				placebo 2/24<br>Sild50 2/21	placebo 0/24<br>Sild50 2/21				placebo 1/24<br>Sild50 1/21					
Becher et al. 2002	Observed and volunteered	most frequent	most mild, transient	placebo 6/71<br>Sild25-100 17/72					placebo 3/71<br>Sild25-100 16/72	placebo 0/71<br>Sild25-100 4/72	placebo 1/71<br>Sild25-100 3/72	placebo 1/71<br>Sild25-100 2/72		
Young et al 2002 Blacks		most common T-R	most mild or mod	placebo 2/122<br>Sild25-100 14/124					placebo 0/122<br>Sild25-100 2/124	placebo 0/122<br>Sild25-100<br>2/124		placebo 0/122<br>Sild25-100<br>2/124		
Young et al 2002 Hispanics		most common T-R	most mild or mod	placebo 7/97<br>Sild25-100 16/98					placebo 2/97<br>Sild25-100 7/98	placebo 0/97<br>Sild25-100 2/98		placebo 1/97<br>Sild25-100 2/98		
Glina et al. 2002		most common T-R	most mild	placebo 4/121<br>Sild25-100 11/124	placebo 0/121<br>Sild25-100 8/124				placebo 0/121<br>Sild25-100<br>11/124	placebo 1/121<br>Sild25-100<br>4/124		placebo 0/121<br>Sild25-100<br>2/124		
Gomez et al. 2002	COSTART	most frequent	most mild	placebo 10/82<br>Sild25-100 19/76	placebo 0/82<br>Sild25-100 5/76				placebo 6/82<br>Sild25-100 9/76	placebo 4/82<br>Sild25-100 4/76	[Palpitation]<br>placebo 2/82<br>Sild25-100 2/76	placebo 1/82<br>Sild25-100 5/76		
Lindsey et al. 2002	Documented	most common	most mild, transient	placebo 0/18<br>Sild25-100 1/14					placebo 2/18<br>Sild25-100 6/14					
Nurnberg et al. 2003	Spontaneous and observed	most common		placebo 4/41<br>Sild25-100 17/43	placebo 0/41<br>Sild25-100 3/43				placebo 1/41<br>Sild25-100 7/43	placebo 2/41<br>Sild25-100 5/43	[Palpitations]<br>placebo 0/41<br>Sild25-100 2/43	placebo 1/41<br>Sild25-100 5/43		
Padma-Nathan et al. 2003		most common	most mild or mod	placebo 0/113<br>Sild100 7/115					placebo 1/113<br>Sild100 4/115			[Sinusitis]<br>placebo 4/113<br>Sild100 0/115		
Kongkanand et al. 2003	Assessed each visit<br>COSTART	all	all mild	placebo 3/62<br>Sild25-100 4/63		placebo 1/62<br>Sild25-100 1/63	placebo 0/62<br>Sild25-100 0/63		placebo 1/62<br>Sild25-100 1/63	placebo 0/62<br>Sild25-100 6/63	placebo 2/62<br>Sild25-100 4/63	placebo 0/62<br>Sild25-100 3/63		placebo 0/62<br>Sild25-100 0/63
Levinson et al. 2003		5%	most mild to mod, transient	placebo 10/126<br>Sild25-100 26/128	placebo 1/126<br>Sild25-100<br>12/128			placebo 9/126<br>Sild25-100<br>5/128	placebo 2/126<br>Sild25-100 8/128	placebo 4/126<br>Sild25-100<br>10/128		placebo 3/126<br>Sild25-100<br>7/128		
Stuckey et al. 2003		5%	all mild to mod, transient	placebo 7/93<br>Sild25-100 19/95	placebo 1/93<br>Sild25-100 8/95				placebo 3/93<br>Sild25-100 17/95	placebo 2/93<br>Sild25-100 2/95				
Choi et al. 2003	Assessed each visit<br>Observed and	at least 3 patients	most mild	placebo 6/67<br>Sild25-100 17/66	placebo 5/67<br>Sild25-100 3/66				placebo 3/67<br>Sild25-100 21/66	placebo 1/67<br>Sild25-100 7/66		placebo 1/67<br>Sild25-100 3/66		placebo 0/67<br>Sild25-100 0/66
Tignol et al. 2004	Observed and volunteered	3%	most mild or mod	placebo 1/85<br>Sild25-100 10/83					placebo 0/85<br>Sild25-100 10/83	placebo 1/85<br>Sild25-100 2/83		placebo 0/85<br>Sild25-100 3/83		
Safarinejad et al. 2004	Recorded at each visit	treatment-related		placebo 3/138<br>Sild100 29/144					placebo 0/138<br>Sild100 27/144		placebo 0/138<br>Sild100 10/144	placebo 0/138<br>Sild100 9/144		placebo 0/138<br>Sild100 0/144
DeBusk et al. 2004		most common	most mild to mod, transient	placebo 1/76<br>Sild25-100 6/74	placebo 4/76<br>Sild25-100 2/74				placebo 0/76<br>Sild25-100 6/74	placebo 1/76<br>Sild25-100 1/74	placebo 3/76<br>Sild25-100 8/74	placebo 0/76<br>Sild25-100 2/74		
Mahon et al. 2005		all		placebo 0/13<br>Sild50-100 1/13	no other AEs reported									
Fowler et al. 2005	Recorded	3%		placebo 8/113<br>Sild25-100 28/104	placebo 0/113<br>Sild25-100 6/104				placebo 2/113<br>Sild25-100<br>13/104	placebo 0/113<br>Sild25-100<br>4/104		placebo 1/113<br>Sild25-100<br>6/104	Weakness<br>placebo 1/113<br>Sild25-100	

Tadalafil trials

Study	Ascertainment and classification	Reporting cut-off level	Severity	Headache	Dyspepsia	Back pain	Myalgia	Flu syndrome	Flushing	Visual disturbances	CV events	Limb pain	Nasal congestion	Fatigue
Padma-Nathan et al 2001	non-leading questions	3%	most mild, transient	Placebo 2/35<br>Tad5 1/37<br>Tad10 6/36<br>Tad20 5/36	Placebo 0/35<br>Tad5 3/37<br>Tad10 1/36<br>Tad20 3/36	Placebo 0/35<br>Tad5 0/37<br>Tad10 3/36<br>Tad20 1/36	Placebo 0/35<br>Tad5 0/37<br>Tad10 1/36<br>Tad20 1/36		1 with Tad	No colour abnormalities				
Saenz de Tejada et al. 2002	All reported COSTART	3%	most mild or mod, transient	Placebo 2/71<br>Tad10 7/73<br>Tad20 6/72	Placebo 0/71<br>Tad10 8/73<br>Tad20 8/72	Placebo 1/71<br>Tad10 1/73<br>Tad20 4/72	Placebo 1/71<br>Tad10 4/73<br>Tad20 3/72	Placebo 3/71<br>Tad10 3/73<br>Tad20 3/72	Placebo 0/71<br>Tad10 2/73<br>Tad20 3/72	No T-R				
Porst et al. 2003	All reported COSTART			Placebo 2/173<br>Tad20 14/175	Placebo 0/173<br>Tad20 9/175	Placebo<br>Tad20	Placebo 0/173<br>Tad20 6/175		Placebo 0/173<br>Tad20 10/175	None	None			
Eardley et al. 2004	All reported MedDRA v5	2%	most mild or mod	Placebo 3/52<br>Tad20 30/168	Placebo 0/52<br>Tad20 22/168	Placebo 0/52<br>Tad20 6/168	Placebo 1/52<br>Tad20 4/168		Placebo 1/52<br>Tad20 8/168	1 blurred vision. No colour abnormalities		Placebo 0/52<br>Tad20 6/168		
Skoumal et al. 2004	Recorded	2%		Placebo 2/104<br>Tad20 22/305		Placebo 1/104<br>Tad20 7/305		Placebo 0/104<br>Tad20 6/305	Placebo 0/104<br>Tad20 14/305				Placebo 0/104<br>Tad20 6/305	
Seftel et al. 2004	Recorded Med DRA	"most common"	most mild or mod	Placebo 3/48<br>Tad20 25/159	Placebo 0/48<br>Tad20 12/159	Placebo 0/48<br>Tad20 14/159					2 with Tad20 had serious chest pain			
Montorsi et al. 2004	Recorded	2%		Placebo 6/102<br>Tad20 42/201	Placebo 1/102<br>Tad2027/201	Placebo 6/102<br>Tad209/201	Placebo 0/102<br>Tad20 13/201		Placebo 0/102<br>Tad20 7/201				Placebo 1/102<br>Tad209/201	Placebo 1/102<br>Tad20 7/201
Carson et al. 2005	Recorded Med DRA	"most common"	most mild or mod	8.9%	4.8%		3.4%							

Vardenafil trials

Study	Ascertainment and classification	Reporting cut-off level	Severity	Headache	Dyspepsia	Back pain	Myalgia	Flu syndrome	Flushing	Visual disturbances	CV events	Limb pain	Rhinitis/Nasal congestion	Fatigue	Hypertension
Porst et al. 2001	Patient recorded	5%		Placebo 6/152<br>Vard5 10/147<br>Vard10 12/141<br>Vard20 23/150	Placebo 0/152<br>Vard5 1/147<br>Vard10 4/141<br>Vard20 10/150				Placebo 1/152<br>Vard5 15/147<br>Vard10 16/141<br>Vard20 17/150	No colour abnormalities<br>6 patients (both gps) had transient inc brightness/haze			Placebo 5/152<br>Vard5 7/147<br>Vard10 4/141<br>Vard20 11/150		
Hellstrom et al.2002	Recorded	5%	most mild or moderate	Placebo 8/182<br>Vard5 19/193<br>Vard10 44/199<br>Vard20 40/188	Placebo 1/182<br>Vard5 2/193<br>Vard10 8/199<br>Vard20 12/188			Placebo 2/182<br>Vard5 10/193<br>Vard10 5/199<br>Vard20 3/188	Placebo 0/182<br>Vard5 9/193<br>Vard10 20/199<br>Vard20 24/188	No blue colour abnormalities<br>No other effect in more than 2%	1 MI with placebo		Placebo 9/182<br>Vard5 17/193<br>Vard10 27/199<br>Vard20 32/188		
Goldstein et al. 2003		5%	most mild or moderate, transient	Placebo 10/143<br>Vard10 20/152<br>Vard20 16/144					Placebo 1/143<br>Vard10 14/152<br>Vard20 14/144	No colour disturbances	1 chest pain, 1 ST depression (group not stated)		Placebo 7/143<br>Vard10 8/152<br>Vard20 15/144		
Brock et al. 2003		5%	most mild or moderate	Placebo 4/140<br>Vard10 16/140<br>Vard20 22/147	Placebo 0/140<br>Vard10 4/140<br>Vard20 5/147										
Hatzichristou et al. 2004	Patient recorded	2%	most mild, transient	Placebo 3/164<br>Vard 18/157	Placebo 1/164<br>Vard 1/157	Placebo 1/164<br>Vard 4/157	[CPK inc]<br>Placebo 3/164<br>Vard 5/157	Placebo 7/164<br>Vard 8/157	Placebo 0/164<br>Vard 19/157				Placebo 4/164<br>Vard 10/157		Placebo 1/164<br>Vard 3/157
Carson et al. 2004	Monitored and reported (visits and phone)	n/a	mostly mild or moderate	no breakdown of AEs provided											
Nagao et al. 2004	Recorded	2%	mild (most) or moderate (10)	Placebo 4/71<br>Vard5 5/67<br>Vard10 9/75<br>Vard20 7/66	Placebo 0/71<br>Vard5 1/67<br>Vard10 0/75<br>Vard20 2/66		[CPK inc]<br>Placebo 0/71<br>Vard5 0/67<br>Vard10 1/75<br>Vard20 2/66		Placebo 4/71<br>Vard5 14/67<br>Vard10 22/75<br>Vard20 24/66		[Palpitation]<br>Placebo 0/71<br>Vard5 3/67<br>Vard10 4/75<br>Vard20 2/66		Placebo 0/71<br>Vard5 3/67<br>Vard10 5/75<br>Vard20 6/66		
